# The Potential Role of Boron in the Modulation of Gut Microbiota Composition: An In Vivo Pilot Study

**DOI:** 10.3390/ph17101334

**Published:** 2024-10-06

**Authors:** Nermin Basak Sentürk, Burcu Kasapoglu, Eray Sahin, Orhan Ozcan, Mehmet Ozansoy, Muzaffer Beyza Ozansoy, Pinar Siyah, Ugur Sezerman, Fikrettin Sahin

**Affiliations:** 1 Department of Genetics and Bioengineering, Faculty of Engineering, Yeditepe University, 34755 Istanbul, Turkey; basak.senturk@yeditepe.edu.tr (N.B.S.); burcu.kasapoglu@abdiibrahim.com.tr (B.K.); 2 Abdi Ibrahim Pharmaceuticals, Biotechnological Products Production Facility (AbdiBio), 34538 Istanbul, Turkey; 3 Biostatistics and Bioinformatics PhD Program, Institute of Health Sciences, Acibadem Mehmet Ali Aydinlar University, 34752 Istanbul, Turkey; sahin.eray89@gmail.com; 4 Cryptomicrobiology, 41400 Kocaeli, Turkey; orhn.ozcn@hotmail.com; 5 Department of Physiology, International School of Medicine, Istanbul Medipol University, 34810 Istanbul, Turkey; mozansoy@medipol.edu.tr; 6 Department of Physical Therapy and Rehabilitation, Faculty of Health Sciences, Fenerbahçe University, 34758 Istanbul, Turkey; beyza.ozansoy@fbu.edu.tr; 7 Department of Biochemistry, School of Pharmacy, Bahçeşehir University, 34353 Istanbul, Turkey; pinar.siyah@med.bau.edu.tr; 8 Department of Biostatistics and Medical Informatics, Faculty of Medicine, Acibadem Mehmet Ali Aydinlar University, 34752 Istanbul, Turkey

**Keywords:** boron derivatives, gut microbiota, obesity, cancer, diabetes, neurodegenerative diseases

## Abstract

**Background/Objectives**: The role of the gut microbiome in the development and progression of many diseases has received increased attention in recent years. Boron, a trace mineral found in dietary sources, has attracted interest due to its unique electron depletion and coordination characteristics in chemistry, as well as its potential role in modulating the gut microbiota. This study investigates the effects of inorganic boron derivatives on the gut microbiota of mice. **Methods**: For three weeks, boric acid (BA), sodium pentaborate pentahydrate (NaB), and sodium perborate tetrahydrate (SPT) were dissolved (200 mg/kg each) in drinking water and administered to wild-type BALB/c mice. The composition of the gut microbiota was analyzed to determine the impact of these treatments. **Results**: The administration of BA significantly altered the composition of the gut microbiota, resulting in a rise in advantageous species such as *Barnesiella* and *Alistipes*. Additionally, there was a decrease in some taxa associated with inflammation and illness, such as *Clostridium XIVb* and *Bilophila*. Notable increases in genera like *Treponema* and *Catellicoccus* were observed, suggesting the potential of boron compounds to enrich microbial communities with unique metabolic functions. **Conclusions**: These findings indicate that boron compounds may have the potential to influence gut microbiota composition positively, offering potential prebiotic effects. Further research with additional analyses is necessary to fully understand the interaction between boron and microbiota and to explore the possibility of their use as prebiotic agents in clinical settings.

## 1. Introduction

Growing emphasis has been given in recent years to the role that the gut microbiome and its metabolites play in the onset and course of many diseases [1,2]. The human gastrointestinal system [3] contains over 100 trillion microbes, the majority of which are bacteria, but there are also viruses, fungi, and protozoa [4], all participating in basic metabolic, physiological, and immune functions [5]; therefore, the human gastrointestinal system can be considered as one of the organs of the human body [6]. The human genome includes roughly 23,000 genes. However, the microbiome has about 3 million genes that produce thousands of metabolites that play roles in numerous host functions, affecting the host’s physical fitness, phenotype, and health in the process [4]. In recent years, there has been a tremendous amount of interest in microorganisms, with the emergence of their potential role in disease pathogenesis and treatment.

The greatest diversity of human bacteria is found in the gut microbiome, especially in the colon [7]. The bioactive substances produced by these gut bacteria affect the general health and illness of the host [8].

Gastrointestinal (GI) inflammation caused by inflammatory bowel disease (IBD) modifies the commensal microbiome [9,10]. Numerous diseases in both humans and animals, such as obesity, allergies, Type I-II diabetes, autism, and colorectal cancer, have also been associated with dysbiosis [11,12]. IBD, which includes Crohn’s disease (CD) and ulcerative colitis (UC), is a GI tract inflammatory disorder that is both chronic and recurrent [13].

Over the past few decades, the gut microbiota has been shown to play a role in promoting weight gain, insulin resistance, and fat storage [14,15]. Indeed, by acquiring energy from food through fermentation and the production of short-chain fatty acids (SCFAs), the gut microbiota contributes to energy homeostasis [16,17]. Changes affecting the dominant phyla *Firmicutes* and *Bacteroidetes* were initially observed in obese animals, and it has been demonstrated that there is a decrease in *Bacteroidetes* accompanied by an increase in *Firmicutes* [18].

In 2011, the first study published by Giongo et al. investigated the association between Type 1 diabetes mellitus (T1DM) and gut microbiota [19]. According to previous research, the gut microbiome of children with T1DM exhibited a lower Shannon diversity and a larger *Bacteroidetes*/*Firmicutes* ratio. The first study on the gut microbiota of Type 2 diabetes mellitus (T2DM) patients in 2010 found a notable decline in *Clostridia* and *Firmicutes* abundance, while *Betaproteobacteria* increased and correlated positively with plasma glucose [20].

Colorectal cancer (CRC) is associated with intestinal dysbiosis, characterised by an elevated presence of potentially pathogenic bacteria and a concurrent decrease in the proportion of butyrate-producing bacteria among CRC patients. Studies have reported diminished production levels of *Proteobacteria*, *Bifidobacteria*, *Prevotella*, and short-chain fatty acids (SCFA), while an increase has been observed in *Firmicutes*, *Bacteroidetes*, *Enterobacteriaceae*, and *Fusobacteria* [11,21].

The intestine has a very close relationship with the central nervous system (CNS). While it connects with the CNS via the sympathetic and parasympathetic nervous systems (Brain–Gut Axis/Connection), the CNS communicates with the intestinal muscle and mucosal layers through autonomic pathways. In this manner, the brain regulates mucus secretion, immunity, permeability, and bowel motions [22]. An increasing number of studies suggest that dysbiosis in the gut microbiota contributes to senescence, oxidative stress, cytokine production, and neuroinflammation in the early phases of Alzheimer’s disease pathogenesis [23]. Recent research found abnormalities in the microbiome of patients with multiple sclerosis (MS), a neuroinflammatory autoimmune disease [24]. Associations with abnormalities in the gut microbiota have also been discovered in psychiatric diseases, such as depression and autism spectrum disorder (ASD) [25,26]. In a study of amyotrophic lateral sclerosis (ALS), a decrease was reported in the density of bacteria that produce butyrate, such as *Butyrivibrio fibrosolvens*, and an increase in serum and intestinal cytokine IL-17 levels in their stool samples [27]. It was discovered that treatment with probiotics such as *Akkermansia muciniphila* ameliorated the symptoms and provided positive results in the course of ALS in SOD1 transgenic mice [28,29].

### 1.1. Boron

Boron is a naturally occurring trace element [30], and for many species, it is classified as a prebiotic playing a crucial role in the origin and development of life and serves as a micronutrient vital for certain bacteria, plants, fungi, and algae [31]. It can be found in healthy tissues as borate or boric acid [32,33]. There are many uses for boron due to its special electron depletion and coordination characteristics in the fields of chemistry, energy research, materials science, and biological sciences [34]. Evidence has accumulated that boron exhibits a variety of pleiotropic actions, including anti-bacterial [35,36,37], anti-inflammatory, and wound-healing actions [38,39,40], as well as modulating other physiological systems. It has been found to be used in several metabolic processes, including bone growth and maintenance [41], hormone activity [42], and mental processes [43,44]. These discoveries have brought attention to the designs of boron-based medicines [45,46].

Many bioactive compounds with boron atoms have been developed over decades [47]. The antioxidant properties of boron compounds are also well-known [48]. The FDA-approved bortezomib (PS-341), also marketed as Velcade, is a dipeptide boronic acid and proteasome inhibitor for the treatment of multiple myeloma [49,50]. Moreover, in a study conducted using a small-cell lung cancer cell model in 2022, the efficacy of derivatives of boric acid (BA), sodium pentaborate pentahydrate (NaB), and sodium perborate tetrahydrate (SPT) was demonstrated to exhibit anti-cancer effects [32]. Tavaborole (AN2690), one of the benzoxaboroles, was FDA-approved in 2014 to treat onychomycosis after it was recognised in 2006 for its antifungal qualities [51,52]. Furthermore, a number of organoboron compounds have potent antibacterial properties, especially against Gram-negative bacteria. The antibiotic oxaborole AN3365/GSK2251052 [53] is one prominent example.

Inorganic boron compounds, like borates and boron clusters, are utilised in therapies such as Boron Neutron Capture Therapy (BNCT) for cancer treatment, leveraging their unique structural and chemical properties to target tumours [54,55]. Some inorganic compounds used in BNCT Therapy are Boronophenylalanine (BPA) and Sodium Borocaptate (BSH).

In the early 1990s, the first potential link between boron, diet, and brain activity was discovered [43]. The first study performed on adult rats revealed that adding more boron to a diet containing less boron improves brain electrical activity [43]. Human studies have been conducted to investigate the effects of boron compounds on brain electrical activity and the performance of various mental and motor activities based on the findings from the previous study [44]. The effects of boron derivatives, boric acid (BA), and sodium borate decahydrate (borax decahydrate) on amyloid beta (A) toxicity were examined in a study conducted by Ozansoy et al. [56]. The results suggest that BA protects against Aβ_1−42_ toxicity in vitro, and it can also be considered a preventive medication thanks to its capacity to boost Sirt1 expression [56]. It has also been confirmed that boron nitride nanotubes (BNNTs) keep peptides of Aβ_1−42_ trimer apart and appear to limit self-aggregation, exhibiting the ability to behave as a therapeutic option for AD treatment [57].

Recent research has shown that boron, when consumed as a dietary supplement, promotes both short- and long-term weight loss [58,59]. In 2017, an in vitro study showed that BA and NaB inhibited adipogenesis by controlling crucial growth factors and suppressed the expressions of the genes associated with adipogenesis [60]. It has also been shown that a diet high in boron significantly reduced the levels of serum total cholesterol, LDL, VLDL, and triglycerides and changed the lipid profile in humans. Additionally, it was demonstrated that body weight, body fat weight, and BMI decreased [61].

### 1.2. Boron and Microbiota: Exploring Links

Boron (B) is an essential element for human host microbiota, and a deficiency of boron can lead to dysbiosis due to a deficiency of the autoinducer-2–furanosyl borate diester (AI-2B) signalling molecule and the degradation of the mucus gel layer in the mucin gel structure, due to a B concentration deficiency in the mucin gel composition, and thereby infections caused by the direct interactions of the bacterial biofilm with the host cell due to an impaired mucus gel layer [31,62,63,64]. These observations helped to create a new perspective regarding the capability of the AI-2B signalling molecule to modulate microbiota [31,65].

Naturally occurring organic boron (NOB) species have emerged as promising prebiotic candidates and have proven to be essential in fostering symbiotic relationships [31,66]. The mechanism of action of NOB species involves acting through the B signalling molecule AI-2B and enhancing the colonic mucus gel layer via their uptake by B-rich diets. Both the microbiota and colonic mucus gel layer are potential targets of NOB, emphasising its significance [31,66,67].

In light of these findings, our study aimed to investigate the potential effects of inorganic boron derivatives on the gut microbiota of wild-type mice as a pilot study. Inspired by the promising prebiotic potential of naturally occurring organic boron (NOB) species, we explored whether certain inorganic boron compounds, known for their pleiotropic health effects, could offer similar benefits. These compounds include NaB (NaB_5_O_8_·5H_2_O), known for its wound-healing (anti-inflammatory) [39], antimicrobial [37], anti-cancer [32], and anti-obesity properties [58,60]. SPT (NaBO_3_·4H_2_O) is recognised for its application as an industrial bleaching agent [68] and its anti-cancer effects [32], and BA (H_3_BO_3_) is found in tissues, exhibits wound-healing (anti-inflammatory) [40], anti-cancer [32], anti-obesity [58,60], and neuroprotective effects in in vitro Alzheimer models [56]. The cytotoxicity assessments for these compounds were performed using in silico techniques. Through our investigation, we sought to elucidate the potential effects of these boron derivatives on the gut microbiota composition in wild-type mice, contributing to the understanding of their biological effects in a preclinical context.

## 2. Results

### 2.1. In Silico Toxicity Assay

The toxicity profiles of the compounds were screened using the eMolTox tool [69] (Tables S5–S7). Predictions were carried out to assess the potential effects of these compounds on critical organs and systems, including the heart, blood, endocrine system, and liver. For the BA compound, predictions indicated that it is unlikely to interact significantly with the Beta-2 adrenergic receptor, suggesting a low likelihood of adverse cardiovascular and hematological effects. Similarly, BA is predicted not to modulate the Vascular Endothelial Growth Factor Receptor (VEGFR), indicating a low cardiovascular health risk. Furthermore, it was found to lack substantial antagonistic effects on the Estrogen Receptor Alpha (ER-α), suggesting a low risk of endocrine disruption. However, predictions related to phospholipidosis induction were inconclusive, and the acute oral toxicity potential of the BA compound also yielded ambiguous results. Analysis of the SPT compound suggested that it does not significantly interact with the Beta-2 adrenergic receptor, indicating a low probability of causing adverse effects on the heart and blood. It was also predicted that VEGFR would not modulate significantly, supporting a favourable cardiovascular safety profile. For the NaB compound, the analysis indicated that it does not significantly interact with the Beta-2 adrenergic receptor, suggesting a low likelihood of adverse cardiovascular and hematological effects. The compound was also predicted to have a low risk of VEGFR modulation, which is positive for cardiovascular health. However, predictions regarding the potential antagonistic effects of both NaB and SPT on Estrogen Receptor Alpha and predictions related to phospholipidosis induction were inconclusive. The docking studies to evaluate the binding of three candidate compounds to the hERG (human ether-à-go-go-related gene) channel were performed [70]. Based on the docking scores (Table S8), the reference compound [71] had a binding affinity score of −4.456 kcal/mol. The resulting binding affinity scores for the candidate compounds were relatively low (NaB: −3.301 kcal/mol, SPT: −2.990 kcal/mol, and BA: −2.904 kcal/mol), suggesting that these compounds are unlikely to inhibit the hERG channel.

### 2.2. Gut Bacterial Diversity in Mice Treated with Different Boron Compounds

**Figure 1:**
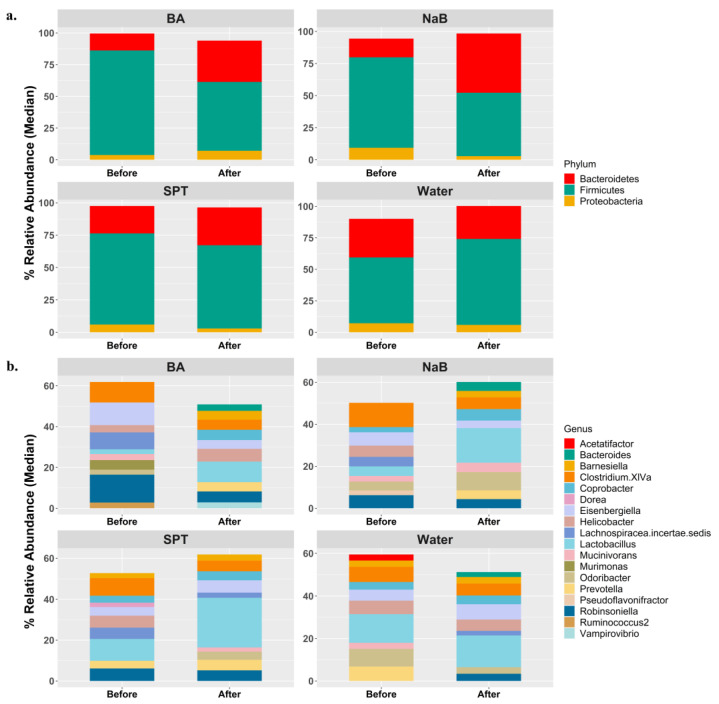
General view of the gut microbiota compositions in each group at the beginning and at the end of treatments based on (**a**) the top 3 phyla and (**b**) the top 10 genera with respect to median relative abundance values. Water (*n* = 5), BA: boric acid (*n* = 4), NaB: sodium pentaborate pentahydrate (*n* = 5), SPT: sodium perborate tetrahydrate (*n* = 4).

Based on a general view of the bacterial composition at the phylum level, *Firmicutes*, *Bacteroidetes*, and Proteobacteria were the three dominant groups in all mice groups both before and after the treatments (Figure 1a). On the other hand, such homogeneity could not be observed in median relative abundance at the genus level (Figure 1b).

**Figure 2:**
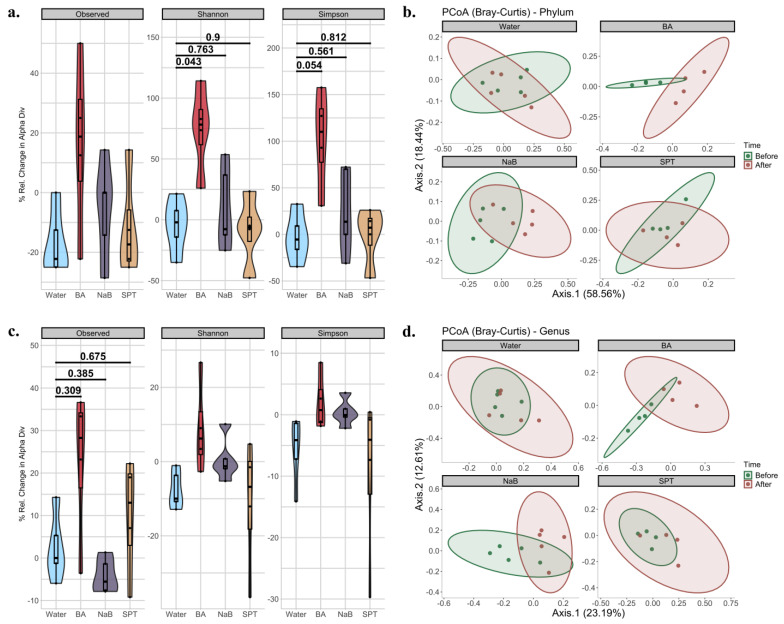
Diversity analysis of mouse intestinal microbiota compositions before and after treatment for each group. (**a**) Changes in the three alpha diversity metrics at the phylum level based on the observed features, Shannon index, and Simpson index; (**b**) PCoA plots of beta diversity in phylum-level compositions before and after treatments based on Bray-Curtis dissimilarity; (**c**) changes in the three alpha diversity metrics at the genus level based on observed features, Shannon index, and Simpson index; (**d**) PCoA plots of beta diversity in genus-level compositions based on Bray-Curtis dissimilarity before and after treatments. BA: boric acid, NaB: sodium pentaborate pentahydrate, SPT: sodium perborate tetrahydrate, and the control group was supplied with water. For alpha diversity, data were represented by mixed violin and box plots for each time point. Subject-specific changes before and after treatment are shown by red, blue, or grey lines to represent increased, decreased, or even values, respectively. Adjusted *p* values for respective pairwise comparisons were added if the respective KW *p* value < 0.1. For beta diversity, the time points were represented by different colours for before and after treatments. Points represent each mouse sample. Ellipses correspond to 95% confidence intervals for each of the treatment groups. Water (*n* = 5), BA: boric acid (*n* = 4), NaB: sodium pentaborate pentahydrate (*n* = 5), SPT: sodium perborate tetrahydrate (*n* = 4).

Alpha and beta diversity metrics are used to explain within and between sample diversity, respectively. In alpha diversity measurements, richness and evenness within a sample are evaluated by counting different features and measuring the distribution of the features based on their relative abundance, respectively [72]. In this study, three metrics were employed to analyse alpha diversity. First, observed features were used to explain bacterial richness only [72]. Secondly, the Shannon diversity index, which takes both richness and evenness into account by providing equal weight to both, was used [72]. Lastly, the Simpson index, another metric examining both richness and evenness like Shannon diversity, was used. Unlike the Shannon index, it gives more weight to evenness [72]. All metrics were calculated separately at the phylum (Figure 2a) and genus (Figure 2c) levels. When the percentage of changes between two time points was calculated, and changes in treatment groups were compared to the changes in the control (water) group in time, and the BA vs. control group changes and comparisons only gave statistical significance at the phylum level for Shannon and Simpson indices (Observed: KW *p* value = 0.249. Shannon: KW *p* value = 0.046; BA vs. Control *p*.adj = 0.043, NaB vs. Control *p*.adj = 0.763, SPT vs. Control *p*.adj = 0.900. Simpson: KW *p* value = 0.049; BA vs. Control *p*.adj = 0.054, NaB vs. Control *p*.adj = 0.561, SPT vs. Control *p*.adj = 0.812.) (Figure 2a). On the other hand, none of the comparisons yielded significance at the genus levels for any of the three indices based on *p*.adj values (Observed: KW *p* value = 0.098; BA vs. Control *p*.adj = 0.309, NaB vs. Control *p*.adj = 0.385, SPT vs. Control *p*.adj = 0.675. Shannon: KW *p* value = 0.102. Simpson: KW *p* value = 0.109).

Beta diversity analysis, which is used to analyse community-level differences between sample diversity, was carried out, and the communities between two time points were statistically tested at both the phylum (Figure 2b, Supplementary Table S2) and genus (Figure 2d, Supplementary Table S2) levels. In phylum-level time comparisons based on Bray–Curtis, there was no significant difference between the control (Water_After vs. Water_Before *p* value = 0.898) and SPT (SPT_After vs. SPT_Before *p* value = 0.425) groups. However, both BA (BA_After vs. BA_Before *p* value = 0.03) and NaB (NaB_After vs. NaB_Before *p* value = 0.018) treatments caused a significant shift in bacterial compositions. In genus-level time comparisons, in agreement with phylum-level results, no significance was obtained from the control (Water_After vs. Water_Before *p* value = 0.158) and SPT (SPT_After vs. SPT_Before *p* value = 0.13) groups, while there was a significant change observed in response to treatment with BA (BA_After vs. BA_Before *p* value = 0.03) or NaB (NaB_After vs. NaB_Before *p* value = 0.027) for Bray–Curtis. Jaccard dissimilarity index-based calculations and analysis also showed similar results (Figure S3, Tables S2 and S4).

### 2.3. Alterations in Gut Microbiota Compositions in Response to Boron 

In order to determine the significant treatment-specific changes compared to the control group, log2 fold changes in each taxonomic group were compared as treatment vs. control at the phylum and genus levels. In phylum-level comparisons, visually, the changes in two of the most dominant phyla, *Bacteroidetes* and *Firmicutes*, were in opposite directions (Figure S1). While *Bacteroidetes* levels increased after administration of boron derivatives, *Firmicutes* levels decreased much more clearly in BA and NaB group subjects. When we tested whether those changes had any significance, timewise comparisons yielded no significance for both phyla (*Firmicutes*: Control_After vs. Control_Before *p*.adj value = 1.00, BA_After vs. BA_Before *p*.adj value = 0.333, NaB_After vs. NaB_Before *p*.adj value = 0.468, SPT_After vs. SPT_Before *p*.adj value = 1.00. *Bacteroidetes*: Control_After vs. Control_Before *p*.adj value = 1.00, BA_After vs. BA_Before *p*.adj value = 0.333, NaB_After vs. NaB_Before *p*.adj value = 0.375, SPT_After vs. SPT_Before *p*.adj value = 0.60). When we compared log2 fold changes between time points for each treatment group and tested the changes in each treatment group compared to the control based on the KW test, *p* values were less than 0.1 for three phyla, *Actinobacteria* (KW *p* value = 0.091), *Bacteroidetes* (KW *p* value = 0.061) and *Proteobacteria* (KW *p* value = 0.076), and *Firmicutes*/*Bacteroidetes* ratio (FB ratio) (KW *p* value = 0.075). However, none of the pairwise comparisons of the changes in each treatment group compared to the control (water) group were revealed as significant (*Actinobacteria*: BA vs. Control *p*.adj = 0.107, NaB vs. Control *p*.adj = 0.597, SPT vs. Control *p*.adj = 0.824. *Bacteroidetes*: BA vs. Control *p*.adj = 0.127, NaB vs. Control *p*.adj = 0.127, SPT vs. Control *p*.adj = 0.845. *Proteobacteria*: BA vs. Control *p*.adj = 0.325, NaB vs. Control *p*.adj = 0.354, SPT vs. Control *p*.adj = 0.625. FB ratio: BA vs. Control *p*.adj = 0.152, NaB vs. Control *p*.adj = 0.152, SPT vs. Control *p*.adj = 0.812).

**Figure 3:**
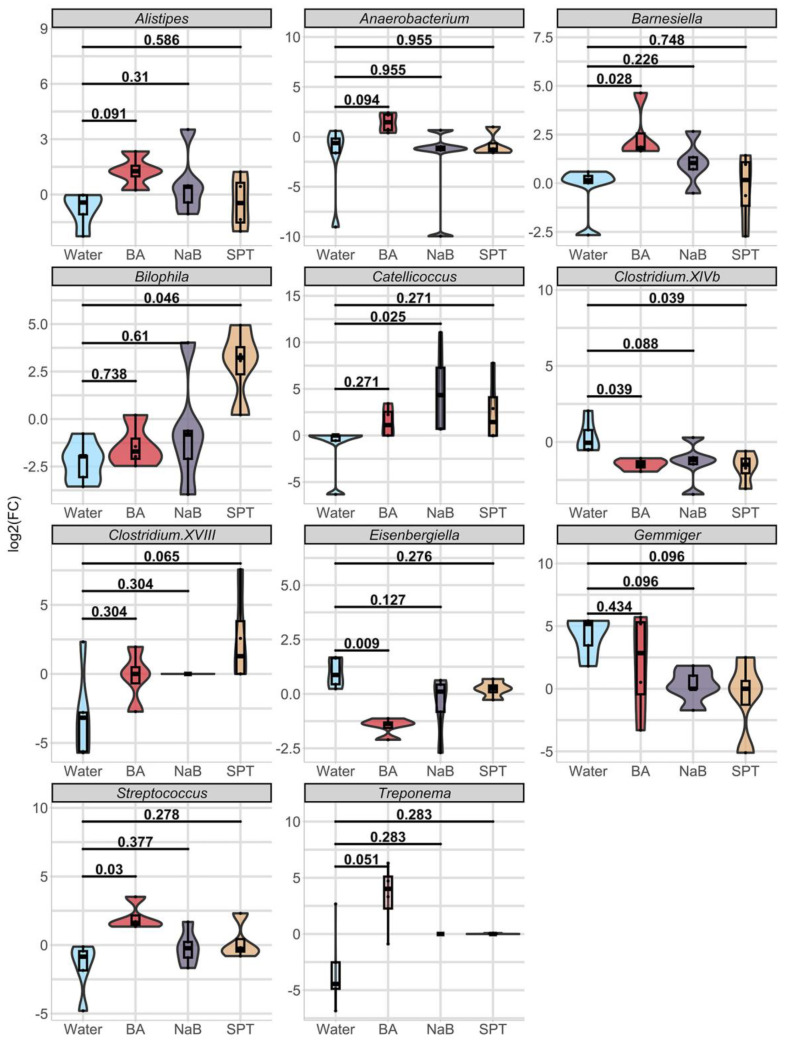
Log2 fold change comparisons of eleven genera in response to each treatment. Data were represented by mixed violin and box plots for each time point. Subject-specific changes before and after treatment are shown by red, blue, or grey lines to represent increased, decreased, or even values, respectively. Adjusted *p* values for respective pairwise comparisons were added if respective KW *p* value < 0.1. Water (*n* = 5), BA: boric acid (*n* = 4), NaB: sodium pentaborate pentahydrate (*n* = 5), SPT: sodium perborate tetrahydrate (*n* = 4).

At the genus level, timewise comparisons of each genus showed no significant difference (Wilcoxon *p*.adj ≥ 0.1) (Figure S4). When we tested the log2 fold changes of each genus in response to treatment compared to changes in the control group, we obtained significant changes in eleven genera (Figure 3, Table S3). Among three treatments vs. water comparisons, the BA treatment caused the most diverse alterations in the mouse gut bacteria genus, as shown by seven of the eleven genera based on statistical analysis from the BA vs. control changes comparison (Figure 3, Table S3). Of these, *Alistipes* (BA log2(FC)_median_ = +1.27, *p*.adj = 0.091), *Anaerobacterium* (BA log2(FC)_median_ = +1.46, *p*.adj = 0.094), *Barnesiella* (BA log2(FC)_median_ = +1.81, *p*.adj = 0.028), *Streptococcus* (BA log2(FC)_median_ = +1.61, *p*.adj = 0.03), and *Treponema* (BA = from 0% relative abundance to a median of 0.029% relative abundance, *p*.adj = 0.051) levels increased with BA; on the other hand, *Costridium XIVb* (BA log2(FC)_median_ = −1.45, *p*.adj = 0.039) and *Eisenbergiella* (BA log2(FC)_median_ = −1.37, *p*.adj = 0.0096) abundance decreased.

NaB treatment caused a significant shift in the abundance of *Clostridium XIVb* and *Catellicoccus* (Figure 3). While the direction change was positive for *Catellicoccus* (NaB = from 0% relative abundance to a median of 0.02% relative abundance, *p*.adj = 0.025), it was negative for *Gemmiger* (NaB log2(FC)median = 0, *p*.adj = 0.096) and *Clostridium XIVb* (NaB log2(FC)median = −1.19, *p*.adj = 0.088).

Finally, SPT treatment enhanced *Bilophila* levels (SPT log2(FC)_median_ = +3.23, *p*.adj = 0.046) and *Clostridium XVIII* levels (SPT log2(FC)_median_ = +1.29, *p*.adj = 0.065) and decreased *Gemmiger* (SPT log2(FC)median = 0, *p*.adj = 0.096) and *Clostridium XIVb* relative abundance (SPT log2(FC)_median_ = −1.49, adjusted *p* value = 0.039) (Table S3).

## 3. Discussion

Commensal bacteria are vital elements of the gut microbiota that are essential to the preservation of human health [73]. Deficient host immune systems, exhibiting innate and adaptive reactions to bacterial encounters, arise from the lack of commensal bacteria [74,75]. This imbalance in intestinal homeostasis also leads to increased gut permeability and intestinal inflammation, which can cause intestinal bacteria to translocate, aggravating the inflammatory response and causing tissue damage [76]. Therefore, commensal bacteria are essential for immune system training and improving infection resistance. Additionally, they have a key role in the synthesis of immunomodulating substances that enhance immune responses, such as short-chain fatty acids (SCFAs), secondary bile acids, amino acids, and vitamins [74,75]. While *Actinobacteria*, *Proteobacteria*, *Fusobacteria*, *Firmicutes*, and *Verrucomicrobia* are the main microbial phyla that make up the gut microbiota, 90% of the gut microbiota is comprised of *Firmicutes* and *Bacteroidetes*. The *Firmicutes* phylum includes genera such as *Lactobacillus*, *Bacillus*, *Enterococcus*, and *Ruminococcus*. *Clostridium* species account for ninety-five percent of the *Firmicutes* phylum [75].

In recent years, the role of boron in modulating microbiota and its potential use as a prebiotic has emerged [31,62,63,64,65,66,67,77]. More recent studies have shown that the signalling molecule containing B in bacteria—furanosyl borate diester, also known as autoinducer-2-borate, or AI-2B—contributes to the health of the animal host through intestinal flora and defence against infections [66,67,78]. Sugar alcohol B esters (SBEs) have also been shown to be potential human health modulators in recent years, similar to NOB species [66,79,80,81]. It has been suggested that SBEs can both decrease virulence in some bacteria and boost their helpful qualities in others [31] due to their resembling AI-2B [66]. In this study, we aimed to investigate the impact of supplementation of three different boron compounds in the gut bacterial composition of wild-type BALB/c mice. NaB, SPT, and BA are the boron derivatives we used that have demonstrated various health benefits, including wound-healing, anti-inflammatory [38,39,40], antimicrobial [35,36,37], anti-cancer [56], anti-obesity [58,60], and neuroprotective effects [56].

The toxicity profiles of the compounds were screened using the eMolTox prediction tool. Predictions were carried out to assess the potential effects of these compounds on critical organs and systems, including the heart, blood, endocrine system, and liver. A combination of statistical classification models and molecular docking experiments was used to evaluate the toxicity profiles of BA, SPT, and NaB. Although the findings indicate that the compounds are unlikely to have a significant effect on the Vascular Endothelial Growth Factor Receptor (VEGFR), which is important for angiogenesis and cardiovascular health, or the Beta-2 adrenergic receptor, which controls breathing and cardiovascular processes, some predictions, including the induction of phospholipidosis and interactions with the Estrogen Receptor Alpha (ER-α), appeared inconclusive. The hERG (human ether-à-go-go-related gene) channel was also given particular consideration. This channel is essential for evaluating drug-induced long QT syndrome, which can result in serious and possibly lethal cardiac arrhythmias. When the hERG channel is inhibited, the electrocardiogram (ECG)’s QT interval is extended, which can lead to torsades de pointes and other serious heart rhythm abnormalities. Positively, docking experiments showed that the likelihood of cardiotoxicity was low due to the low binding affinities of BA, SPT, and NaB to the hERG channel. Nonetheless, the inconclusive predictions regarding phospholipidosis and ER-α interactions emphasise the necessity for more thorough assessments. In statistical classification models, an outcome is “inconclusive” when both active (p1) and inactive (p0) predicted probabilities exceed or fall below a set threshold (ε), indicating insufficient similarity to training set compounds or structural uncertainty [69]. This may explain the ambiguity in phospholipidosis and ER-α interactions, highlighting the need for more precise molecular assessments. These findings imply that additional research may be needed to elucidate the structural characteristics of these compounds in order to determine their potential for toxicity, especially concerning liver health and endocrine disruption. These results provide early information about the safety profiles of the compounds based on a pilot study. In order to provide more reliable data on their behaviour in biological systems, future research should overcome uncertainties through in vivo toxicity assessments.

In our study, boron application through gastric gavage has specific effects on the bacterial populations in gut microbiota. The microbial diversity within and between the samples was assessed using alpha and beta diversity indices. Three metrics were used to measure alpha diversity at the phylum and genus levels: the observable features and the Shannon and Simpson indices. When comparing the BA treatment group to the control, notable variations were seen at the phylum level, especially for the Shannon (*p*.adj = 0.043) and Simpson (*p*.adj = 0.054) indices. None of the measurements, however, revealed statistically significant differences between the groups at the genus level. Bray–Curtis dissimilarity was utilised to evaluate community-level variations in beta diversity. At the phylum and genus levels, there were significant changes in the bacterial composition after treatments with BA (*p* = 0.03) and NaB (*p* = 0.018). In contrast, there were no significant changes at the phylum level in the control (*p* = 0.898) or SPT-treated (*p* = 0.425) groups. Similarly, significant changes were seen for BA (*p* = 0.03) and NaB (*p* = 0.027) at the genus level but not for control or SPT. Similar findings from the Jaccard dissimilarity index study supported the notion that BA and NaB treatments significantly alter microbial diversity.

Two dominant phyla, *Bacteroidetes* and *Firmicutes*, levels changed in opposite directions for all the treatment groups. Even though BA and NaB administration decreased *Firmicutes* and increased *Bacteroidetes*, both changes were not determined to be significant. We also tested the changes in the *Firmicutes/Bacteroidetes* ratio, which is widely used as an obesity indicator [82]. Despite that *p*.adj values are slightly above for NaB and BA, considering the small size of the study, it is noteworthy that supplementation of those two derivatives caused a reduction in the *Firmicutes/Bacteroidetes* ratio. In this context, as mentioned by Doğan et al., it may be considered that BA, in conjunction with other utilised boron derivatives, possesses potential anti-obesity properties. However, further investigations with a larger sample size are necessary to confirm these initial findings more precisely. *Actinobacteria* were mostly missing in many subjects but still survived in the filtering procedure. The abundance of *Actinobacteria* increased in the treatment groups, especially in the BA-treated group; however, no significant variations were found between the treatment groups and the control group. The *Actinobacteria* phylum is a regular member of gut microbiota in both mice and humans. Numerous studies have revealed the beneficial roles of Actinobacteria members, including the biodegradation of certain molecules, immune maturation and modulation, and probiotic features [83].

A total of eleven genera were found to be significantly altered in response to the different boron derivative treatments, most of which differences were determined to be associated with BA. Only one genus responded negatively to all three boron derivative treatments, *Clostridium XIVb*. The relative abundances of the *Alistipes*, *Anaerobacterium*, *Barnesiella*, *Streptococcus*, and *Treponema* genera significantly increased in BA-treated mice compared to the differences in the control group. Conversely, *Eisenbergiella* levels dropped after BA treatment. Significant changes in the bacterial composition were also seen after the NaB treatment; *Catellicoccus* abundance increased, and *Clostridium XIVb* and *Gemmiger* levels decreased. On the other hand, SPT treatment increased the amount of *Clostridium XVIII* and *Bilophila* and reduced the amount of *Gemmiger* and *Clostridium XIVb.*

The relative abundance of the *Barnesiella* genus increased significantly with BA treatment, compared to the control group. *Barnesiella* is an anaerobic genus under the *Bacteroidetes* phylum and *Barnesiellaceae* family and plays a role in short-chain fatty acid (SCFA) production [84]. *Barnesiella* was been one of the most annotated taxa in a study where a subset of the healthy faecal samples collected for the Human Microbiome Project was re-analysed to identify novel taxonomic groups of human gut microbiota [85]. It has also been listed as one of the mouse core microbiota members and as critical for immune regulation [86]. To date, several beneficial roles of the bacteria belonging to the *Barnesiella* genus have been revealed. In one of those early studies, a negative correlation between gut *Barnesiella* abundance and vancomycin-resistant *Enterococcus faecium* colonisation has been shown, and its potential role in treatment against these antibiotics-resistant bacteria was suggested [87]. Elevated *Barnesiella* levels were reported in the treatment of T2DM using a traditional Chinese herb, Xiexin Tang, which is correlated with increased caecal faecal iso-butyric acid and butyric acid concentrations in rat models [88]. Moreover, the protective role of *Barnesiella* against dextran sulfate sodium-induced colitis in respective mouse models has been shown [87,88,89,90]. On the other hand, there are also other findings associating higher *Barnesiella* levels with disease study groups. For example, of the gut microbiota compositions of the faecal samples from healthy controls and two patient groups with Parkinson’s Disease with normal cognitive ability or mild cognitive loss, *Barnesiella* genera levels were found to be higher in patients with mild cognitive loss [91]. In another study, higher levels of *Barnesiella* bacteria but lower butyrate concentrations were reported in children diagnosed with autism spectrum disorders accompanied by constipation [92].

The second genus found to be increased with respect to BA treatment was *Alistipes*. *Alistipes* are anaerobic bacteria, which were identified after an analysis of human clinical samples in 2003, and members of the *Bacteroidetes* phylum [93]. They are healthy mouse core microbiota members, and their levels have been reported to increase as the mouse gets older, contrary to our observations in the water group [86]. Animal-based protein-rich diets have been shown to enhance *Alistipes* abundance in gut microbiota and correlate with increased branched-chain fatty acid concentrations [94,95]. *Alistipes* bacteria have also been associated with the production of some SCFAs like propionate and acetate [96]. Research carried out on mice fed with a high-fat diet revealed the production of sphingolipids, which had been shown to have different beneficial properties such as anti-inflammatory properties, through two gut community members, *Alistipes* and *Odoribacter* [97,98]. Conflicting findings define the role of the *Alistipes* species as a member of gut microbiota. Several members of this genus have been linked with the prevention of illnesses like liver fibrosis, gut inflammation, and cancer [96]. As one example, higher levels of *Alistipes* have been reported in healthy gut microbiota compared to patients diagnosed with non-alcoholic fatty liver disease [99]. One of the current reports revealed the protective role of one species belonging to *Alistipes* genus, *Alistipes putredinis*, against *Candida albicans* colonisation in the human gut [100].

Another member of this genus, *Alistipes finegoldii*, has been implicated in protection for dextran sulfate sodium-induced colitis in mouse subjects [101]. Contrarily, elevated levels of *Alistipes* bacteria and pathogenic effects have been associated with the promotion of several diseases such as autism, mood disorders, cancer, and cardiovascular diseases [96]. Correlations with opposite effects in the same pathologies highlight the necessity of further detailed analyses to reveal the exact role and mechanism behind the development and maintenance of health or disease status.

*Streptococcus* was another genus whose density increased as a result of the BA treatment. It is a genus of facultative anaerobic bacteria and one of the first inhabitants in the establishment of the gut microbiota, which are then replaced by other bacteria, such as the hostages [102]. In a large cohort study, there was a direct relationship between the quantity of *Streptococcus* and joint inflammation and pain in the knee [103]. A study carried out on infants to investigate the impact of gut microbiota on atopic dermatitis has established a positive correlation between *Streptococcus* abundance and disease severity and pointed out the possible involvement in the establishment of dysbiosis in early childhood [104]. A correlation between the relative abundance of *Streptococcus* in the upper intestinal mucosa microbiome and functional dyspepsia has been proposed in a study carried out by Fukui and colleagues [105]. One of the species belonging to the *Streptococcus* genus, *Streptococcus gallolyticus* subsp. *Gallolyticus*, has been classified as an opportunistic pathogen and linked with several pathologies, including bacteremia, infective endocarditis, and colorectal cancer [106]. Despite such pathogenic roles, another species of this genus, *S. thermophilus*, has been shown to exhibit several beneficial effects in diseases like T2DM, inflammation, and sepsis and is used as a prebiotic supplementation [107,108,109]. A species-level sequencing for *Streptococcus* could provide clearer insight into the potential effects of its derivatives on the microbiota.

The abundance of members within the *Anaerobacterium* genus showed a significant rise exclusively in the BA treatment group. The genus was discovered through analysis of soil samples in 2014 [110]. Analyses of stool samples from mice and rats from two different studies have shown that having a high-fat diet elevates *Anaerobacterium*, highlighting potential involvement in diet-based obesity development [111,112]. It has also been listed among non-persistent genera in probiotics supplementation [113]. One of the current studies in Alzheimer’s disease (AD) to investigate the relationship between gut microbiota and clinical scores regarding cognitive functions and neuropsychiatric symptoms, besides reduced abundance in AD patients compared to healthy controls, *Anaerobacterium* levels have been correlated with better cognitive status and less neuropsychiatric symptoms in AD patients [114]. Higher taxonomic resolution down to strain level through shotgun metagenomics could provide clearer insights into the effects of the derivative supplementation on gut microbiome composition within the context of the gut–brain axis in neurodegenerative diseases.

Three of the eight genera, namely, *Clostridium XIVb*, *Eisenbergiella*, and *Bilophila*, were found to be less abundant with respect to the boric acid derivative supplementations.

*Clostridium* is a genus of anaerobic bacteria under the *Firmicutes* phylum [115]. One of the clusters within this genus, *Clostridium XIVb*, has been proposed to be classified as a new genus called *Tyzzerella* [116]. Hence, the following literature information was retrieved using both the *Clostridium XIb* and *Tyzzerella* genus names. *Clostridium XIVb* abundance levels were further decreased in mice treated with all boron derivatives compared to the levels in the control mice group. *Clostridiium XIVb* has been listed among bacteria with elevated levels in the gut microbiota of patients with Rett’s Syndrome compared to healthy controls, which is a neurological disease with comorbidities related to gastrointestinal functions [117]. *Tyzzerella* abundance has been linked to higher cardiovascular disease [118]. On the other hand, in comparison to esophageal cancer and healthy control subjects, *Tyzzerella* bacteria has been detected at higher levels in the control group [119]. Gut microbiota composition analysis in cirrhotic patients has pointed out that the presence of *Ruminococcus* and *Clostridium XIVb* might be associated with better cognitive function in these patients [120]. An increase is reported in the abundance of opportunistic pathogenic bacteria (*Clostridium hathewayi*, *Clostridium symbiosum*, and *Clostridium ramosum*) in T2DM patients [121]. *Clostridium XVIII* is a genus whose relative abundance is significantly reduced by SPT. Intestinal failure was interestingly associated with decreased counts of some *Clostridium* species in clusters *III, IV*, and *XIVa* [122]. Furthermore, these *Clostridium* species are thought to be excellent options for treating intestinal dysfunctions. After taking 17 strains of *Clostridium* clusters *IV*, *XIVa*, and *XVIII* orally, the symptoms of colitis and allergic diarrhoea were shown to improve [123]. It was discovered that these strains enhanced the growth and differentiation of Treg cells, which lessened colitis and allergic diarrhoea in mice. Crucially, none of the strains possessed genes linked to virulence, like phospholipase C or collagenase, which are often present in pathogenic *Clostridia* species [123,124]. Therefore, the increase in *Clostridium XVIII* abundance following SPT treatment may be beneficial, as this genus has been associated with the alleviation of intestinal disorders such as colitis and allergic diarrhoea.

Another genus whose relative abundance of members decreased significantly with BA treatment was *Eisenbergiella*, compared to the control group. *Eisenbergiella* was first identified in a blood sample of a patient in 2014 and shown to produce several SCFAs, including butyrate, lactate, acetate, and succinate [125]. Two *Eisenbergiella* species, *E. tayi* and *E. massiliensis*, have been observed in higher abundance in fibromyalgia patients compared to healthy individuals [126]. Another possible beneficial involvement of *Eisenbergiella* bacteria has been described in a study, and a lower abundance has been associated with chronic intestinal inflammation [127]. Another study has shown decreased *Eisenbergiella* levels in high-fat- and high-sucrose-fed and obesity-induced mice [128]. *Eisenbergiella* has been listed among disrupted genera in functional dyspepsia as being in reduced abundance compared to healthy control in rats [129]. Notably, elevated *Eisenbergiella* levels have been shown in *Echinococcus granulosus* infection and are associated with a Th2 response [130].

*Bilophila* is one of the genera determined to be significantly changing with SPT treatment; its relative abundance elevated after SPT supplementation compared to the control group. *Bilophila* genus was first identified with *Bilophila wadsworthia* species obtained from specimens collected from patient appendicitis and faecal samples in 1989 [131]. It is an anaerobic, nitrate-reducing bacterium with urease and catalase activities and belongs to the *Proteobacteria* phylum [131,132]. A detailed study carried out on mice with high fat intake has revealed that this diet regimen causes an increase in *B. wadsworthia* abundance, which will result in elevated inflammation and gut permeability, decreased butanoate metabolism and butyrate production, a disruption in glucose metabolism and hepatic homeostasis, dysregulations in bile acid metabolism and increased taurine-conjugated bile acid concentrations [133]. Taurine metabolism activity and hydrogen sulfide production in these bacteria provide more evidence of their role in inflammation [134]. In parallel to these findings, the pathogenic potential of these bacteria has also been shown in a study carried out by Feng et al.; when they transferred *B. wadsworthia* obtained from faecal samples of patients with latent autoimmune diabetes to healthy mice, decreased weight and fat mass and elevated immune signatures were reported [135]. Apart from inflammatory properties, obesity-related gut microbiota research carried out on children with different weight profiles has revealed a higher *Bilophila* abundance in the obese group [136].

One of the genera whose relative abundance dropped after receiving both NaB and SPT treatments is *Gemmiger.* It was discovered that, in comparison to infants with the gastrointestinal bleeding type of Henoch–Schonlein Purpura (HSP), children with the renal damage type had a much higher abundance of *Gemmiger* (the genus to species levels). Furthermore, in children with HSP, the abundance of *Gemmiger* was positively linked with serum levels of IL-6, IgA, and IgG, indicating a possible connection between *Gemmiger* and the immune system in this disease [137]. Research has shown that, when it comes to inflammatory bowel illnesses (IBD), such as Crohn’s disease (CD) and ulcerative colitis (UC), healthy individuals usually have larger Gemmiger abundances than IBD patients. This implies that Gemmiger may be a component of a balanced gut microbiota and that a decrease in it may be linked to a dysbiotic state in IBD [138]. Therefore, a shift towards a dysbiotic gut environment may be indicated by the reduction in Gemmiger by NaB and SPT boron compounds; however, this has to be read within the larger framework of the composition and function of the entire microbiome. To completely comprehend the therapeutic ramifications of these variations in Gemmiger levels, more investigation is required.

Treponema is defined to be a part of the ancient human gut microbiome and can still be observed in rural community members with a non-Western diet [139]. Treponema succinifaciens is a known carbohydrate metaboliser in swine. Referring to their roles in the swine microbiome, the genus may also be involved in carbohydrate metabolism [139].

These results have shown that BA induces a significant increase in the *Treponema* genus. *Treponema* (*Treponema pallidum* ssp. *pallidum*) is primarily known for its association with syphilis [140], but different species within the genus can also be part of the normal microbiota in certain body sites. The importance of these bacteria in health and disease continues to be explored in scientific research.

*Catellicoccus* bacteria are facultative anaerobic organisms and are widely found in the gut microbiota of gulls [141]. In a study on humans to investigate the impacts of Ramadan fasting, a positive association has been defined between *Catelliococcus* and the consumption of sweets [142]. In the group treated with NaB, an increase in the relative abundance of the *Catellicoccus* genus has been observed, making it the only genus in this study to exhibit a significant increase in response to the NaB derivative.

*Lactobacillus*, a Gram-positive genus, is prevalent in the gastrointestinal tract and fermented foods [143]. It constitutes about 6.0% of the duodenum’s and 0.3% of the colon’s bacterial populations [144]. These bacteria can attach to the intestinal lining and produce antimicrobial substances like ethanol, hydrogen peroxide, acetic acid, lactic acid, and succinic acid, helping to inhibit potentially harmful bacteria [145]. Certain *Lactobacillus* strains may boost the immune system by activating macrophages, NK cells, and cytotoxic T-lymphocytes, along with promoting cytokine release. Additionally, they can enhance gut immunity by increasing IgA(+) cell recruitment [144]. Although the adjusted *p* values exceed the threshold, it is noteworthy that an increase in the relative abundance of the *Lactobacillus* genus, which is known for its rather beneficial properties, was observed across all groups treated with boron. Enhancing the sample size in future studies could enable the acquisition of clearer outcomes within this context. 

Based on our results, our investigation of boron supplementation’s effect on the gut microbiota composition in BALB/c mice provides important new information about the possible therapeutic advantages of this dietary intervention. However, in order to support our conclusions, a number of limitations must be addressed in future studies, including the small sample size and short study duration. Additionally, the mechanisms behind the observed changes in the gut microbiota should be investigated. Indeed, although notable alterations were noted in specific bacterial taxa, the exact processes responsible for these modifications are yet unknown. There are various possible mechanisms that could mediate these effects. First of all, boron compounds such as boric acid have the ability to change the pH of the surrounding environment [146], which may increase the growth of beneficial bacteria while suppressing the growth of harmful bacteria. Furthermore, boron has the ability to affect the metabolic pathways of bacteria, hence affecting the synthesis of vital metabolites like short-chain fatty acids (SCFAs) [64], which are critical for gut health. Boron has also been shown to have immunomodulatory properties, which may change the immunological environment in the gut and, thus, indirectly influence the diversity of the gut microbiota [147]. A healthy gut microbiota may also be promoted by some boron compounds that function as prebiotics [31,62,63,64,65,66,67,77], supporting the growth of beneficial bacteria like *Bifidobacterium* and *Lactobacillus* [31]. These findings demonstrate boron’s potential as a gut microbiota modulator and imply that the mechanistic relations between the boron and the bacterial genera showing alterations must be clarified further. Our study might be considered an initial step in elucidating these mechanistic relations.

In particular, the consumption of boric acid (BA) revealed significant alterations in the gut microbiota, including rises in advantageous genera such as *Barnesiella* and *Alistipes*, which are connected to various health advantages like the synthesis of short-chain fatty acids and possible anti-obesity properties. These beneficial bacterial alterations imply that BA, in particular, deserves more research as a potential prebiotic. Therefore, future research should focus on determining the precise processes by which boron compounds affect gut microbiota, with a particular emphasis on their possible prebiotic benefits, in light of the results of this pilot study. Research using cutting-edge omics technologies, including metabolomics and metatranscriptomics, may shed light on how boron affects the metabolic processes and gene expression of microorganisms, thereby pointing to new areas for therapeutic intervention. However, larger experimental groups and species-specific sequencing are also necessary to comprehend the underlying mechanisms of boron–microbiota interactions fully. Consequently, it may also be possible to determine the role of boron in avoiding inflammatory and metabolic illnesses by doing long-term research that evaluates the effects of continuous supplementation during various life stages and health conditions. Furthermore, due to boron’s diverse biological activity, it is also important to investigate how it interacts with other dietary elements, such as fibre or polyphenols, in order to maximise its health-promoting qualities and create comprehensive dietary plans. Understanding these pathways is essential for fully utilising boron and its derivatives in therapeutic contexts for the prevention and treatment of disorders associated with gut dysbiosis.

## 4. Materials and Methods

### 4.1. Ethical Approval

All animal procedures were conducted in accordance with the guidelines approved by the Animal Experiments Local Ethics Committee of Istanbul Medipol University ((İMÜ-HADYEK, Istanbul, Turkey) (10 October 2019/70)).

### 4.2. Mouse Model Handling and Boron Application

Wild-type BALB/c mice were bred in our facility, and 6-week-old male mice were preferred for the study in order to eliminate any uncertainties linked to the gender-based variations. They were fed ad libitum with the standard chow diet with constant access to water under a 12:12 h light/dark cycle as previously described [148]. Mice were randomly divided into four groups, each consisting of at least four animals, with some groups containing five animals. All groups were treated with boron derivatives dissolved in their drinking water. Sodium pentaborate pentahydrate (NaB), boric acid (BA), or sodium perborate tetrahydrate (SPT) was administered to mice by gavage at 200 mg/kg every other day for three weeks. The dosage was adapted from the work of Ozansoy et al. (2020) [56]. Only water was given to the control groups (Table 1). Faecal samples were collected immediately before the first and after the last boron treatment.

### 4.3. DNA Isolation, Library Preparation, and Sequencing

All fresh faecal samples were stored at −80 °C after collection. For DNA isolation, up to 200 mg of faecal sample was processed using a ZymoBIOMICS DNA Miniprep Kit (Zymo Research, Orange, CA, USA, Cat. No. D4300). DNA concentrations were measured using a Qubit dsDNA HS Assay Kit (Invitrogen, San Diego, CA, USA, Cat. No. Q32854). The library preparation was carried out using a 16S barcoding kit (Oxford Nanopore Technologies, Oxford, UK, Cat. No. SQK-RAB204) following the manufacturer’s guidelines (version RAB_9053_v1_revL_14Aug2019). Prepared libraries were loaded to R9.4.1 FLO-MIN 106 flow cells, and sequencing was performed on a MinION sequencer (Oxford Nanopore Technologies).

### 4.4. In Silico Analysis

The molecular names were retrieved from PubChem, and the corresponding SMILES codes were input into the prediction tool for a toxicity assessment. In silico docking studies were conducted using methodologies consistent with those in our prior research [149]. Briefly, the hERG channel structure was obtained from the Protein Data Bank (PDB) with the access code 5VA1. Missing loops and side chains within the channel structure were meticulously reconstructed using the Prime module [150], ensuring a complete and accurate representation. The structure was then prepared under physiological conditions, specifically at a pH of 7.4, to simulate the human body’s environment accurately. Comprehensive optimisations and energy minimisations were carried out to achieve an energetically favourable and biologically relevant conformation. The protein was prepared for docking using Maestro’s Glide module, and the active binding site was identified based on the literature [71], focusing on key amino acid residues essential for channel function. A grid box was established around these critical residues to allow precise docking analysis. The ligands were subjected to detailed optimisation procedures, which included maintaining a physiological pH of 7.4, followed by energy minimisation to simulate physiological conditions accurately. The hERG blocker CHEMBL1257698 [71] was used as the reference compound for evaluation. Subsequently, these optimised ligands were docked into the predefined grid box, and their binding affinities were calculated.

### 4.5. Bioinformatics and Statistical Analysis

Raw FAST5 reads were converted to fastq format using Guppy (ver. 6.0.5). The primer sequence removal from amplicon reads and quality trimming was carried out by BBTools (ver. 38.94) [151]. Reads passing quality filtering were clustered by magicblast (ver. 1.6.0) [152] using the reference of 16S rRNA regions of bacteria associated with the human microbiome by the Human Microbiome Project. The consensus sequences were created, and .sam files were produced by Samtools (ver. 1.12) [153]. Taxonomic annotations were determined through BLAST (ver. 2.12.0) in the NCBI nr database, using a sequence identity threshold of 95% for the genus level. Finally, relative abundance percentages were calculated at the phylum and genus levels. All microbiota analyses were carried out at the taxonomic levels of phylum and genus. All data analysis steps were carried out in R (version 4.1). The Phyloseq package (version 1.36) was used to calculate alpha and beta diversity metrics. Before diversity calculations, a rarefaction was performed with a rarefaction depth of 51,662. The significance of the changes in alpha diversity metrics was assessed using the Kruskal-Wallis H (KW) test, followed by a post hoc Dunn’s test with Benjamini-Hochberg correction, and an adjusted *p* value of 0.1 was used as a threshold for both the KW *p* value and *p*.adjusted value from Dunn’s test. Beta diversity analysis was carried out based on the Bray-Curtis dissimilarity index, and statistical significances of the differences between time points were checked using the adonis function in the vegan package (version 2.5.7) [154]. For significant changes between groups at the phylum and genus levels, count data were transformed into relative abundance percentage values, and taxa with a relative abundance of less than 0.0001% in more than 20% of all samples were filtered out before downstream analysis. The differences in bacterial abundances before and after treatments were quantified as log2 fold changes for each treatment group. The calculated fold changes between the treatment and control (water) groups were tested using the KW test, followed by a post hoc Dunn’s test with Benjamini-Hochberg correction, and an adjusted *p* value of 0.1 was used as a significance threshold.

## 5. Conclusions

The importance of the gut microbiota in human health and illness is widely acknowledged. Our study highlights the potential therapeutic benefits of boron supplementation by offering important insights into the impact of boron compounds on the composition of the gut microbiota in BALB/c mice. In particular, the administration of boric acid (BA) showed noteworthy changes in the gut microbiota, including notable increases in beneficial genera like *Barnesiella* and *Alistipes*, which are linked to a number of health benefits, including the production of short-chain fatty acids and potential anti-obesity effects. Although there were no significant changes in the *Firmicutes/Bacteroidetes* ratio in any of the treatment groups, the observed decreases point to the possibility that boron compounds may have an impact on metabolic health and obesity markers. In this study, the gut microbiome of mice is examined in relation to dietary supplements that contain boric acid (BA) and its derivatives. Our research indicates that these substances may alter the gut flora’s composition, causing some genera—such as *Streptococcus*, *Anaerobacterium*, and *Treponema*—to increase and other genera—such as *Clostridium XIVb* (*Tyzzerella*), *Eisenbergiella*, and *Bilophila*—to decrease. All of these changes could have an impact on the health of the host; they include the harmful relationships between *Streptococcus* and *Bilophila* as well as the advantageous properties of *Anaerobacterium* and possibly even *Lactobacillus*, even if the latter’s increase did not achieve statistical significance. The decline in disease-causing taxa, like *Clostridium XIVb* and *Bilophila*, in reaction to BA treatments may suggest a positive adjustment of the intestinal environment, lowering the risk or intensity of illness. Additionally, although the precise roles of these taxa in the context of boron supplementation are still unclear, the rise in *Treponema* and *Catellicoccus* highlights the potential for boron compounds to enrich microbial communities that may have distinct metabolic functions or health implications. While these modifications could potentially enhance general health outcomes, we believe future studies, including larger experimental groups, longer study durations, species-specific sequencing, and advanced omics technologies, are necessary to gain a deeper comprehension of the fundamental mechanisms underlying boron-microbiota interactions in various disease conditions.

Understanding these mechanisms is crucial in order to fully utilise boron and its derivatives for the prevention and treatment of disorders linked to gut dysbiosis as well as to investigate their potential application as prebiotic agents in clinical settings.

## Figures and Tables

**Table 1:** Characteristics of the mice groups used in this study.

Groups	Species of Animal	Substance to Be Applied
Group I (*n* = 5)	BALB/c wild type	Control (water)
Group II (*n* = 4)	BALB/c wild type	Sodium pentaborate pentahydrate (NaB)
Group III (*n* = 4)	BALB/c wild type	Sodium perborate Tetrahydrate (SPT)
Group IV (*n* = 5)	BALB/c wild type	Boric acid (BA)

## Data Availability

The datasets generated and analysed during the current study are available from the corresponding author upon reasonable request.

## Supplementary Materials

The following supporting information can be downloaded at https://www.mdpi.com/article/10.3390/ph17101334/s1, Table S1: Phylum Level Taxonomic Analysis; Table S2: Genus Level Taxonomic Analysis; Table S3: Genus DA Analysis Results; Table S4: Genus Diversity Analysis Results; Table S5: Toxicity predictions of BA; Table S6: Toxicity predictions of NaB; Table S7: Toxicity predictions of SPT; Table S8: Binding affinity predictions of boron derivatives to hERG channel; Figure S1. Time-wise changes in relative abundance of Firmicutes, Bacteroidetes, and in Firmicutes/Bacteroidetes (FB) ratio for each treatment group; Figure S2. Time-wise changes in observed features, Shannon and Simpson indices in each treatment group for genus level; Figure S3. PCoA plots of beta diversity in (a) phylum and (b) genus level compositions based on Jaccard dissimilarity before and after treatments; Figure S4. Time-wise changes in relative abundance of the eleven genera in each treatment group.

## References

[1] Wu J., Wang K., Wang X., Pang Y., Jiang C. (2021). The Role of the Gut Microbiome and Its Metabolites in Metabolic Diseases. Protein Cell.

[2] Okubo H., Nakatsu Y., Kushiyama A., Yamamotoya T., Matsunaga Y., Inoue M.-K., Fujishiro M., Sakoda H., Ohno H., Yoneda M. (2018). Gut Microbiota as a Therapeutic Target for Metabolic Disorders. Curr. Med. Chem..

[3] Beaumont M., Goodrich J.K., Jackson M.A., Yet I., Davenport E.R., Vieira-Silva S., Debelius J., Pallister T., Mangino M., Raes J. (2016). Heritable Components of the Human Fecal Microbiome Are Associated with Visceral Fat. Genome Biol..

[4] Valdes A.M., Walter J., Segal E., Spector T.D. (2018). Role of the Gut Microbiota in Nutrition and Health. BMJ.

[5] Baquero F., Nombela C. (2012). The Microbiome as a Human Organ. Clin. Microbiol. Infect..

[6] Hirschberg S., Gisevius B., Duscha A., Haghikia A. (2019). Implications of Diet and the Gut Microbiome in Neuroinflammatory and Neurodegenerative Diseases. Int. J. Mol. Sci..

[7] Sender R., Fuchs S., Milo R. (2016). Revised Estimates for the Number of Human and Bacteria Cells in the Body. PLoS Biol..

[8] Conlon M.A., Bird A.R. (2014). The Impact of Diet and Lifestyle on Gut Microbiota and Human Health. Nutrients.

[9] Khan I., Ullah N., Zha L., Bai Y., Khan A., Zhao T., Che T., Zhang C. (2019). Alteration of Gut Microbiota in Inflammatory Bowel Disease (IBD): Cause or Consequence? IBD Treatment Targeting the Gut Microbiome. Pathogens.

[10] Shan Y., Lee M., Chang E.B. (2022). The Gut Microbiome and Inflammatory Bowel Diseases. Annu. Rev. Med..

[11] DeGruttola A.K., Low D., Mizoguchi A., Mizoguchi E. (2016). Current Understanding of Dysbiosis in Disease in Human and Animal Models. Inflamm. Bowel Dis..

[12] Zhang Y.J., Li S., Gan R.Y., Zhou T., Xu D.P., Li H.B. (2015). Impacts of Gut Bacteria on Human Health and Diseases. Int. J. Mol. Sci..

[13] Kochar B., Orkaby A.R., Ananthakrishnan A.N., Ritchie C.S. (2021). Frailty in Inflammatory Bowel Diseases: An Emerging Concept. Therap. Adv. Gastroenterol..

[14] Cardinelli C.S., Sala P.C., Alves C.C., Torrinhas R.S., Waitzberg D.L. (2015). Influence of Intestinal Microbiota on Body Weight Gain: A Narrative Review of the Literature. Obes. Surg..

[15] Magne F., Gotteland M., Gauthier L., Zazueta A., Pesoa S., Navarrete P., Balamurugan R. (2020). The *Firmicutes/Bacteroidetes* Ratio: A Relevant Marker of Gut Dysbiosis in Obese Patients?. Nutrients.

[16] Jumpertz R., Le D.S., Turnbaugh P.J., Trinidad C., Bogardus C., Gordon J.I., Krakoff J. (2011). Energy-Balance Studies Reveal Associations between Gut Microbes, Caloric Load, and Nutrient Absorption in Humans. Am. J. Clin. Nutr..

[17] Turnbaugh P.J., Ley R.E., Mahowald M.A., Magrini V., Mardis E.R., Gordon J.I. (2006). An Obesity-Associated Gut Microbiome with Increased Capacity for Energy Harvest. Nature.

[18] Ley R.E., Turnbaugh P.J., Klein S., Gordon J.I. (2006). Human Gut Microbes Associated with Obesity. Nature.

[19] Giongo A., Gano K.A., Crabb D.B., Mukherjee N., Novelo L.L., Casella G., Drew J.C., Ilonen J., Knip M., Hyöty H. (2011). Toward Defining the Autoimmune Microbiome for Type 1 Diabetes. ISME J..

[20] Larsen N., Vogensen F.K., Van Den Berg F.W.J., Nielsen D.S., Andreasen A.S., Pedersen B.K., Al-Soud W.A., Sørensen S.J., Hansen L.H., Jakobsen M. (2010). Gut Microbiota in Human Adults with Type 2 Diabetes Differs from Non-Diabetic Adults. PLoS ONE.

[21] Akbar N., Khan N.A., Muhammad J.S., Siddiqui R. (2022). The Role of Gut Microbiome in Cancer Genesis and Cancer Prevention. Heal. Sci. Rev..

[22] Belkaid Y., Hand T.W. (2014). Role of the Microbiota in Immunity and Inflammation. Cell.

[23] Leblhuber F., Steiner K., Geisler S., Fuchs D., Gostner J.M. (2020). On the Possible Relevance of Bottom-up Pathways in the Pathogenesis of Alzheimer’s Disease. Curr. Top. Med. Chem..

[24] Chen J., Chia N., Kalari K.R., Yao J.Z., Novotna M., Soldan M.M.P., Luckey D.H., Marietta E.V., Jeraldo P.R., Chen X. (2016). Multiple Sclerosis Patients Have a Distinct Gut Microbiota Compared to Healthy Controls. Sci. Rep..

[25] Kang D.W., Park J.G., Ilhan Z.E., Wallstrom G., LaBaer J., Adams J.B., Krajmalnik-Brown R. (2013). Reduced Incidence of *Prevotella* and Other Fermenters in Intestinal Microflora of Autistic Children. PLoS ONE.

[26] Jiang H., Ling Z., Zhang Y., Mao H., Ma Z., Yin Y., Wang W., Tang W., Tan Z., Shi J. (2015). Altered Fecal Microbiota Composition in Patients with Major Depressive Disorder. Brain. Behav. Immun..

[27] Wu S., Yi J., Zhang Y.G., Zhou J., Sun J. (2015). Leaky Intestine and Impaired Microbiome in an Amyotrophic Lateral Sclerosis Mouse Model. Physiol. Rep..

[28] Zhang Y.G., Wu S., Yi J., Xia Y., Jin D., Zhou J., Sun J. (2017). Target Intestinal Microbiota to Alleviate Disease Progression in Amyotrophic Sclerosis. Clin. Ther..

[29] Blacher E., Bashiardes S., Shapiro H., Rothschild D., Mor U., Dori-Bachash M., Kleimeyer C., Moresi C., Harnik Y., Zur M. (2019). Potential Roles of Gut Microbiome and Metabolites in Modulating ALS in Mice. Nature.

[30] World Health Organization, International Atomic Energy Agency, Food and Agriculture Organization of the United Nations (1996). Trace Elements in Human Nutrition and Health.

[31] Biţă A., Scorei I.R., Bălşeanu T.A., Ciocîlteu M.V., Bejenaru C., Radu A., Bejenaru L.E., Rău G., Mogoşanu G.D., Neamţu J. (2022). New Insights into Boron Essentiality in Humans and Animals. Int. J. Mol. Sci..

[32] Cebeci E., Yüksel B., Şahin F. (2022). Anti-Cancer Effect of Boron Derivatives on Small-Cell Lung Cancer. J. Trace Elem. Med. Biol..

[33] Devirian T., Volpe S. (2003). The Physiological Effects of Dietary Boron. Crit. Rev. Food Sci. Nutr..

[34] Angenent P. (2023). Advances in Boron-based Drugs in Medicinal Chemistry. Arch. Chem. Res. Adv. Boron-based Drugs Med. Chem..

[35] Brittingham A., Wilson W.A. (2014). The Antimicrobial Effect of Boric Acid on *Trichomonas vaginalis*. Sex. Transm. Dis..

[36] Trippier P., McGuigan C. (2010). Boronic Acids in Medicinal Chemistry: Anticancer, Antibacterial and Antiviral Applications. Medchemcomm.

[37] Demirci S., Kaya M.S., Doğan A., Kalay Ş., Altin N.Ö., Yarat A., Akyüz S.H., Şahin F. (2015). Antibacterial and Cytotoxic Properties of Boron-Containing Dental Composite. Turk. J. Biol..

[38] Demirci S., Doğan A., Karakuş E., Halıcı Z., Topçu A., Demirci E., Sahin F. (2015). Boron and Poloxamer (F68 and F127) Containing Hydrogel Formulation for Burn Wound Healing. Biol. Trace Elem. Res..

[39] Doğan A., Demirci S., Çağlayan A.B., Kılıç E., Günal M.Y., Uslu Ü., Cumbul A., Şahin F. (2014). Sodium Pentaborate Pentahydrate and Pluronic Containing Hydrogel Increases Cutaneous Wound Healing in Vitro and in Vivo. Biol. Trace Elem. Res..

[40] Coskun M. (2023). Success in Treating Wounds with Local Boric Acid: A Case Study. J. Wound Care.

[41] Nielsen F.H. (2014). Update on Human Health Effects of Boron. J. Trace Elem. Med. Biol..

[42] Sheng M.H., Janette Taper L., Veit H., Qian H., Ritchey S.J., William Lau K.H. (2001). Dietary Boron Supplementation Enhanced the Action of Estrogen, but Not That of Parathyroid Hormone, to Improve Trabecular Bone Quality in Ovariectomized Rats. Biol. Trace Elem. Res..

[43] Penland J.G. (1994). Dietary Boron, Brain Function, and Cognitive Performance. Environ. Health Perspect..

[44] Penland J.G. (1998). The Importance of Boron Nutrition for Brain and Psychological Function. Biol. Trace Elem. Res..

[45] Fernandes G., Denny W., Dos Santos J. (2019). Boron in Drug Design: Recent Advances in the Development of New Therapeutic Agents. Eur. J. Med. Chem..

[46] Das B.C., Thapa P., Karki R., Schinke C., Das S., Kambhampati S., Banerjee S.K., Van Veldhuizen P., Verma A., Weiss L.M. (2013). Boron Chemicals in Diagnosis and Therapeutics. Future Med. Chem..

[47] Baker S.J., Ding C.Z., Akama T., Zhang Y.-K., Hernandez V., Xia Y. (2009). Therapeutic Potential of Boron-Containing Compounds. Future Med. Chem..

[48] Barranco W.T., Kim D.H., Stella S.L., Eckhert C.D. (2009). Boric Acid Inhibits Stored Ca^2+^ Release in DU-145 Prostate Cancer Cells. Cell Biol. Toxicol..

[49] Adams J., Behnke M., Chen S., Cruickshank A.A., Dick L.R., Grenier L., Klunder J.M., Ma Y.-T., Plamondon L., Stein R.L. (1998). Potent and Selective Inhibitors of the Proteasome: Dipeptidyl Boronic Acids. Bioorg. Med. Chem. Lett..

[50] Scorei R., Popa R. (2010). Boron-Containing Compounds as Preventive and Chemotherapeutic Agents for Cancer. Anticancer. Agents Med. Chem..

[51] Adamczyk-Wozniak A., Borys K.M., Sporzynski A. (2015). Recent Developments in the Chemistry and Biological Applications of Benzoxaboroles. Chem. Rev..

[52] Sharma N., Sharma D. (2015). An Upcoming Drug for Onychomycosis: Tavaborole. J. Pharmacol. Pharmacother..

[53] Zhang P., Ma S. (2019). Recent Development of Leucyl-TRNA Synthetase Inhibitors as Antimicrobial Agents. Medchemcomm.

[54] Ali F., S Hosmane N., Zhu Y. (2020). Boron Chemistry for Medical Applications. Molecules.

[55] Teixidor F., Núñez R., Viñas C. (2023). Towards the Application of Purely Inorganic Icosahedral Boron Clusters in Emerging Nanomedicine. Molecules.

[56] Ozansoy M., Altintaş M., Ozansoy M., Günay N., Kiliç E., Kiliç Ü. (2020). Two Boron-Containing Compounds Affect the Cellular Viability of SH-SY5Y Cells in an in Vitro Amyloid-Beta Toxicity Model. Turk. J. Biol..

[57] Ciofani G., Del Turco S., Genchi G.G., D’Alessandro D., Basta G., Mattoli V. (2012). Transferrin-Conjugated Boron Nitride Nanotubes: Protein Grafting, Characterization, and Interaction with Human Endothelial Cells. Int. J. Pharm..

[58] Aysan E., Sahin F., Telci D., Erdem M., Muslumanoglu M., Yardmc E., Bektasoglu H. (2013). Mechanism of Body Weight Reducing Effect of Oral Boric Acid Intake. Int. J. Endocrinol..

[59] Kucukkurt I., Akbel E., Karabag F., Ince S. (2015). The Effects of Dietary Boron Compounds in Supplemented Diet on Hormonal Activity and Some Biochemical Parameters in Rats. Toxicol. Ind. Health.

[60] Doğan A., Demirci S., Apdik H., Faruk Bayrak O., Gulluoglu S., Can Tuysuz E., Gusev O., Rizvanov A.A., Nikerel E., Şahin F. (2017). A New Hope for Obesity Management: Boron Inhibits Adipogenesis in Progenitor Cells through the Wnt/β-Catenin Pathway. Metabolism.

[61] Kuru R., Yilmaz S., Balan G., Tuzuner B.A., Tasli P.N., Akyuz S., Yener Ozturk F., Altuntas Y., Yarat A., Sahin F. (2019). Boron-Rich Diet May Regulate Blood Lipid Profile and Prevent Obesity: A Non-Drug and Self-Controlled Clinical Trial. J. Trace Elem. Med. Biol..

[62] Biţă A., Scorei I.R., Rangavajla N., Bejenaru L.E., Rău G., Bejenaru C., Ciocîlteu M.V., Dincă L., Neamţu J., Bunaciu A. (2023). Diester Chlorogenoborate Complex: A New Naturally Occurring Boron-Containing Compound. Inorganics.

[63] Mitruţ I., Scorei I.R., Manolea H.O., Biţă A., Mogoantă L., Neamţu J., Bejenaru L.E., Ciocîlteu M.V., Bejenaru C., Rău G. (2022). Boron-Containing Compounds in Dentistry: A Narrative Review. Rom. J. Morphol. Embryol. = Rev. Roum. Morphol. Embryol..

[64] Biţă C.E., Scorei I.R., Vreju A.F., Muşetescu A.E., Mogoşanu G.D., Biţă A., Dinescu V.C., Dinescu Ş.C., Criveanu C., Bărbulescu A.L. (2023). Microbiota-Accessible Boron-Containing Compounds in Complex Regional Pain Syndrome. Medicina.

[65] Thompson J.A., Oliveira R.A., Djukovic A., Ubeda C., Xavier K.B. (2015). Manipulation of the Quorum Sensing Signal AI-2 Affects the Antibiotic-Treated Gut Microbiota. Cell Rep..

[66] Hunter J.M., Nemzer B.V., Rangavajla N., Biţă A., Rogoveanu O.C., Neamţu J., Scorei I.R., Bejenaru L.E., Rău G., Bejenaru C. (2019). The Fructoborates: Part of a Family of Naturally Occurring Sugar–Borate Complexes—Biochemistry, Physiology, and Impact on Human Health: A Review. Biol. Trace Elem. Res..

[67] Donoiu I., Militaru C., Obleagă O., Hunter J.M., Neamţu J., Biţă A., Scorei I.R., Rogoveanu O.C. (2018). Effects of Boron-Containing Compounds on Cardiovascular Disease Risk Factors—A Review. J. Trace Elem. Med. Biol. Organ Soc. Miner. Trace Elem..

[68] Attin T., Paqué F., Ajam F., Lennon Á.M. (2003). Review of the Current Status of Tooth Whitening with the Walking Bleach Technique. Int. Endod. J..

[69] Ji C., Svensson F., Zoufir A., Bender A. (2018). EMolTox: Prediction of Molecular Toxicity with Confidence. Bioinformatics.

[70] Onder F.C., Siyah P., Durdagi S., Ay M., Ozpolat B. (2022). Novel Etodolac Derivatives as Eukaryotic Elongation Factor 2 Kinase (EEF2K) Inhibitors for Targeted Cancer Therapy. RSC Med. Chem..

[71] Creanza T.M., Delre P., Ancona N., Lentini G., Saviano M., Mangiatordi G.F. (2021). Structure-Based Prediction of HERG-Related Cardiotoxicity: A Benchmark Study. J. Chem. Inf. Model..

[72] Wagner B.D., Grunwald G.K., Zerbe G.O., Mikulich-Gilbertson S.K., Robertson C.E., Zemanick E.T., Harris J.K. (2018). On the Use of Diversity Measures in Longitudinal Sequencing Studies of Microbial Communities. Front. Microbiol..

[73] Lee J., Song X., Hyun B., Jeon C.O., Hyun S. (2023). Drosophila Gut Immune Pathway Suppresses Host Development-Promoting Effects of Acetic Acid Bacteria. Mol. Cells.

[74] McCuaig B., Goto Y. (2023). Immunostimulating Commensal Bacteria and Their Potential Use as Therapeutics. Int. J. Mol. Sci..

[75] Rinninella E., Raoul P., Cintoni M., Franceschi F., Miggiano G.A.D., Gasbarrini A., Mele M.C. (2019). What Is the Healthy Gut Microbiota Composition? A Changing Ecosystem across Age, Environment, Diet, and Diseases. Microorganisms.

[76] Nong K., Qin X., Liu Z., Wang Z., Wu Y., Zhang B., Chen W., Fang X., Liu Y., Wang X. (2024). Potential Effects and Mechanism of Flavonoids Extract of *Callicarpa nudiflora* Hook on DSS-Induced Colitis in Mice. Phytomedicine.

[77] Hsiao A., Ahmed A.M.S., Subramanian S., Griffin N.W., Drewry L.L., Petri W.A.J., Haque R., Ahmed T., Gordon J.I. (2014). Members of the Human Gut Microbiota Involved in Recovery from *Vibrio cholerae* Infection. Nature.

[78] Thompson J.A., Oliveira R.A., Xavier K.B. (2016). Chemical Conversations in the Gut Microbiota. Gut Microbes.

[79] Miljkovic D., Scorei R.I., Cimpoiaşu V.M., Scorei I.D. (2009). Calcium Fructoborate: Plant-Based Dietary Boron for Human Nutrition. J. Diet. Suppl..

[80] Hu Y., Quan H., Cui J., Luo W., Zeng W., Chen D. (2021). Carbon Nanodot Modified N, O-Doped Porous Carbon for Solid-State Supercapacitor: A Comparative Study with Carbon Nanotube and Graphene Oxide. J. Alloys Compd..

[81] Kim J., Oh J., Lee K.Y., Jung I., Park M. (2017). Dispersion of Graphene-Based Nanocarbon Fillers in Polyamide 66 by Dry Processing and Its Effect on Mechanical Properties. Compos. Part B Eng..

[82] Stojanov S., Berlec A., Štrukelj B. (2020). The Influence of Probiotics on the *Firmicutes/Bacteroidetes* Ratio in the Treatment of Obesity and Inflammatory Bowel Disease. Microorganisms.

[83] Binda C., Lopetuso L.R., Rizzatti G., Gibiino G., Cennamo V., Gasbarrini A. (2018). *Actinobacteria*: A Relevant Minority for the Maintenance of Gut Homeostasis. Dig. Liver Dis. Off. J. Ital. Soc. Gastroenterol. Ital. Assoc. Study Liver.

[84] Zhou L., Xiao X., Zhang Q., Zheng J., Li M., Wang X., Deng M., Zhai X., Liu J. (2019). Gut Microbiota Might Be a Crucial Factor in Deciphering the Metabolic Benefits of Perinatal Genistein Consumption in Dams and Adult Female Offspring. Food Funct..

[85] Wylie K.M., Truty R.M., Sharpton T.J., Mihindukulasuriya K.A., Zhou Y., Gao H., Sodergren E., Weinstock G.M., Pollard K.S. (2012). Novel Bacterial Taxa in the Human Microbiome. PLoS ONE.

[86] Wang J., Lang T., Shen J., Dai J., Tian L., Wang X. (2019). Core Gut Bacteria Analysis of Healthy Mice. Front. Microbiol..

[87] Ubeda C., Bucci V., Caballero S., Djukovic A., Toussaint N.C., Equinda M., Lipuma L., Ling L., Gobourne A., No D. (2013). Intestinal Microbiota Containing *Barnesiella* Species Cures Vancomycin-Resistant *Enterococcus faecium* Colonization. Infect. Immun..

[88] Wei X., Tao J., Xiao S., Jiang S., Shang E., Zhu Z., Qian D., Duan J. (2018). Xiexin Tang Improves the Symptom of Type 2 Diabetic Rats by Modulation of the Gut Microbiota. Sci. Rep..

[89] Weiss G.A., Chassard C., Hennet T. (2014). Selective Proliferation of Intestinal *Barnesiella* under *Fucosyllactose* Supplementation in Mice. Br. J. Nutr..

[90] Li M., Wu Y., Hu Y., Zhao L., Zhang C. (2018). Initial Gut Microbiota Structure Affects Sensitivity to DSS-Induced Colitis in a Mouse Model. Sci. China. Life Sci..

[91] Ren T., Gao Y., Qiu Y., Jiang S., Zhang Q., Zhang J., Wang L., Zhang Y., Wang L., Nie K. (2020). Gut Microbiota Altered in Mild Cognitive Impairment Compared With Normal Cognition in Sporadic Parkinson’s Disease. Front. Neurol..

[92] Liu S., Li E., Sun Z., Fu D., Duan G., Jiang M., Yu Y., Mei L., Yang P., Tang Y. (2019). Altered Gut Microbiota and Short Chain Fatty Acids in Chinese Children with Autism Spectrum Disorder. Sci. Rep..

[93] Rautio M., Eerola E., Väisänen-Tunkelrott M.-L., Molitoris D., Lawson P., Collins M.D., Jousimies-Somer H. (2003). Reclassification of *Bacteroides putredinis* (Weinberg et Al., 1937) in a New Genus *Alistipes* Gen. Nov., as *Alistipes putredinis* Comb. Nov., and Description of *Alistipes finegoldii* sp. Nov., from Human Sources. Syst. Appl. Microbiol..

[94] David L.A., Maurice C.F., Carmody R.N., Gootenberg D.B., Button J.E., Wolfe B.E., Ling A.V., Devlin A.S., Varma Y., Fischbach M.A. (2014). Diet Rapidly and Reproducibly Alters the Human Gut Microbiome. Nature.

[95] Shortt C., Hasselwander O., Meynier A., Nauta A., Fernández E.N., Putz P., Rowland I., Swann J., Türk J., Vermeiren J. (2018). Systematic Review of the Effects of the Intestinal Microbiota on Selected Nutrients and Non-Nutrients. Eur. J. Nutr..

[96] Parker B.J., Wearsch P.A., Veloo A.C.M., Rodriguez-Palacios A. (2020). The Genus Alistipes: Gut Bacteria With Emerging Implications to Inflammation, Cancer, and Mental Health. Front. Immunol..

[97] Walker A., Pfitzner B., Harir M., Schaubeck M., Calasan J., Heinzmann S.S., Turaev D., Rattei T., Endesfelder D., Castell W.Z. (2017). Sulfonolipids as Novel Metabolite Markers of *Alistipes* and *Odoribacter* Affected by High-Fat Diets. Sci. Rep..

[98] Wang X., Wang Y., Xu J., Xue C. (2021). Sphingolipids in Food and Their Critical Roles in Human Health. Crit. Rev. Food Sci. Nutr..

[99] Jiang W., Wu N., Wang X., Chi Y., Zhang Y., Qiu X., Hu Y., Li J., Liu Y. (2015). Dysbiosis Gut Microbiota Associated with Inflammation and Impaired Mucosal Immune Function in Intestine of Humans with Non-Alcoholic Fatty Liver Disease. Sci. Rep..

[100] Mirhakkak M.H., Schäuble S., Klassert T.E., Brunke S., Brandt P., Loos D., Uribe R.V., Senne de Oliveira Lino F., Ni Y., Vylkova S. (2021). Metabolic Modeling Predicts Specific Gut Bacteria as Key Determinants for Candida Albicans Colonization Levels. ISME J..

[101] Dziarski R., Park S.Y., Kashyap D.R., Dowd S.E., Gupta D. (2016). Pglyrp-Regulated Gut Microflora *Prevotella falsenii*, *Parabacteroides distasonis* and *Bacteroides eggerthii* Enhance and *Alistipes finegoldii* Attenuates Colitis in Mice. PLoS ONE.

[102] Bharadia L., Agrawal N., Joshi N. (2020). Development and Functions of the Infant Gut Microflora: Western vs. Indian Infants. Int. J. Pediatr..

[103] Boer C.G., Radjabzadeh D., Medina-Gomez C., Garmaeva S., Schiphof D., Arp P., Koet T., Kurilshikov A., Fu J., Ikram M.A. (2019). Intestinal Microbiome Composition and Its Relation to Joint Pain and Inflammation. Nat. Commun..

[104] Park Y.M., Lee S.Y., Kang M.J., Kim B.S., Lee M.J., Jung S.S., Yoon J.S., Cho H.J., Lee E., Yang S.I. (2020). Imbalance of Gut *Streptococcus*, *Clostridium*, and *Akkermansia* Determines the Natural Course of Atopic Dermatitis in Infant. Allergy. Asthma Immunol. Res..

[105] Fukui A., Takagi T., Naito Y., Inoue R., Kashiwagi S., Mizushima K., Inada Y., Inoue K., Harusato A., Dohi O. (2020). Higher Levels of *Streptococcus* in Upper Gastrointestinal Mucosa Associated with Symptoms in Patients with Functional Dyspepsia. Digestion.

[106] Isenring J., Köhler J., Nakata M., Frank M., Jans C., Renault P., Danne C., Dramsi S., Kreikemeyer B., Oehmcke-Hecht S. (2018). *Streptococcus gallolyticus* subsp. *Gallolyticus endocarditis* Isolate Interferes with Coagulation and Activates the Contact System. Virulence.

[107] Asemi Z., Zare Z., Shakeri H., Sabihi S.-S., Esmaillzadeh A. (2013). Effect of Multispecies Probiotic Supplements on Metabolic Profiles, Hs-CRP, and Oxidative Stress in Patients with Type 2 Diabetes. Ann. Nutr. Metab..

[108] Del Carmen S., de Moreno de LeBlanc A., Martin R., Chain F., Langella P., Bermúdez-Humarán L.G., LeBlanc J.G. (2014). Genetically Engineered Immunomodulatory *Streptococcus thermophilus* Strains Producing Antioxidant Enzymes Exhibit Enhanced Anti-Inflammatory Activities. Appl. Environ. Microbiol..

[109] Han F., Wu G., Zhang Y., Zheng H., Han S., Li X., Cai W., Liu J., Zhang W., Zhang X. (2020). *Streptococcus thermophilus* Attenuates Inflammation in Septic Mice Mediated by Gut Microbiota. Front. Microbiol..

[110] Horino H., Fujita T., Tonouchi A. (2014). Description of *Anaerobacterium chartisolvens* gen. nov., sp. nov., an Obligately Anaerobic Bacterium from *Clostridium* RRNA Cluster III Isolated from Soil of a Japanese Rice Field, and Reclassification of *Bacteroides cellulosolvens* Murray et al. 1984 as *Pseudobacteroides cellulosolvens* gen. nov., comb. nov. Int. J. Syst. Evol. Microbiol..

[111] Deshpande N.G., Saxena J., Pesaresi T.G., Carrell C.D., Ashby G.B., Liao M.-K., Freeman L.R. (2019). High Fat Diet Alters Gut Microbiota but Not Spatial Working Memory in Early Middle-Aged Sprague Dawley Rats. PLoS ONE.

[112] Zhang J., Yi C., Han J., Ming T., Zhou J., Lu C., Li Y., Su X. (2020). Novel High-Docosahexaenoic-Acid Tuna Oil Supplementation Modulates Gut Microbiota and Alleviates Obesity in High-Fat Diet Mice. Food Sci. Nutr..

[113] Ma C., Huo D., You Z., Peng Q., Jiang S., Chang H., Zhang J., Zhang H. (2020). Differential Pattern of Indigenous Microbiome Responses to Probiotic *Bifidobacterium lactis* V9 Consumption across Subjects. Food Res. Int..

[114] Zhou Y., Wang Y., Quan M., Zhao H., Jia J. (2021). Gut Microbiota Changes and Their Correlation with Cognitive and Neuropsychiatric Symptoms in Alzheimer’s Disease. J. Alzheimers. Dis..

[115] Cruz-Morales P., Orellana C.A., Moutafis G., Moonen G., Rincon G., Nielsen L.K., Marcellin E. (2019). Revisiting the Evolution and Taxonomy of *Clostridia*, a Phylogenomic Update. Genome Biol. Evol..

[116] Yutin N., Galperin M.Y. (2013). A Genomic Update on Clostridial Phylogeny: Gram-Negative Spore Formers and Other Misplaced Clostridia. Environ. Microbiol..

[117] Strati F., Cavalieri D., Albanese D., De Felice C., Donati C., Hayek J., Jousson O., Leoncini S., Pindo M., Renzi D. (2016). Altered Gut Microbiota in Rett Syndrome. Microbiome.

[118] Kelly T.N., Bazzano L.A., Ajami N.J., He H., Zhao J., Petrosino J.F., Correa A., He J. (2016). Gut Microbiome Associates With Lifetime Cardiovascular Disease Risk Profile Among Bogalusa Heart Study Participants. Circ. Res..

[119] Deng Y., Tang D., Hou P., Shen W., Li H., Wang T., Liu R. (2021). Dysbiosis of Gut Microbiota in Patients with Esophageal Cancer. Microb. Pathog..

[120] Bajaj J.S., Fagan A., White M.B., Wade J.B., Hylemon P.B., Heuman D.M., Fuchs M., John B.V., Acharya C., Sikaroodi M. (2019). Specific Gut and Salivary Microbiota Patterns Are Linked With Different Cognitive Testing Strategies in Minimal Hepatic Encephalopathy. Am. J. Gastroenterol..

[121] Wang J., Qin J., Li Y., Cai Z., Li S., Zhu J., Zhang F., Liang S., Zhang W., Guan Y. (2012). A Metagenome-Wide Association Study of Gut Microbiota in Type 2 Diabetes. Nat. 2012 4907418.

[122] Till H., Castellani C., Moissl-Eichinger C., Gorkiewicz G., Singer G. (2015). Disruptions of the Intestinal Microbiome in Necrotizing Enterocolitis, Short Bowel Syndrome, and Hirschsprung’s Associated Enterocolitis. Front. Microbiol..

[123] Atarashi K., Tanoue T., Oshima K., Suda W., Nagano Y., Nishikawa H., Fukuda S., Saito T., Narushima S., Hase K. (2013). Treg Induction by a Rationally Selected Mixture of *Clostridia* Strains from the Human Microbiota. Nature.

[124] Guo P., Zhang K., Ma X., He P. (2020). *Clostridium* Species as Probiotics: Potentials and Challenges. J. Anim. Sci. Biotechnol..

[125] Amir I., Bouvet P., Legeay C., Gophna U., Weinberger A. (2014). *Eisenbergiella tayi* gen. nov., sp. nov., Isolated from Human Blood. Int. J. Syst. Evol. Microbiol..

[126] Minerbi A., Gonzalez E., Brereton N.J.B., Anjarkouchian A., Dewar K., Fitzcharles M.-A., Chevalier S., Shir Y. (2019). Altered Microbiome Composition in Individuals with Fibromyalgia. Pain.

[127] Chen Y.-J., Wu H., Wu S.-D., Lu N., Wang Y.-T., Liu H.-N., Dong L., Liu T.-T., Shen X.-Z. (2018). *Parasutterella*, in Association with Irritable Bowel Syndrome and Intestinal Chronic Inflammation. J. Gastroenterol. Hepatol..

[128] Lacroix S., Pechereau F., Leblanc N., Boubertakh B., Houde A., Martin C., Flamand N., Silvestri C., Raymond F., Di Marzo V. (2019). Rapid and Concomitant Gut Microbiota and Endocannabinoidome Response to Diet-Induced Obesity in Mice. mSystems.

[129] Luo L., Hu M., Li Y., Chen Y., Zhang S., Chen J., Wang Y., Lu B., Xie Z., Liao Q. (2018). Association between Metabolic Profile and Microbiomic Changes in Rats with Functional Dyspepsia. RSC Adv..

[130] Bao J., Zheng H., Wang Y., Zheng X., He L., Qi W., Wang T., Guo B., Guo G., Zhang Z. (2018). *Echinococcus granulosus* Infection Results in an Increase in *Eisenbergiella* and *Parabacteroides* Genera in the Gut of Mice. Front. Microbiol..

[131] Baron E.J., Summanen P., Downes J., Roberts M.C., Wexler H., Finegold S.M. (1989). *Bilophila wadsworthia*, Gen. Nov. and Sp. Nov., a Unique Gram-Negative Anaerobic Rod Recovered from Appendicitis Specimens and Human Faeces. J. Gen. Microbiol..

[132] Schoenborn L., Abdollahi H., Tee W., Dyall-Smith M., Janssen P.H. (2001). A Member of the Delta Subgroup of *Proteobacteria* from a Pyogenic Liver Abscess Is a Typical Sulfate Reducer of the Genus *Desulfovibrio*. J. Clin. Microbiol..

[133] Natividad J.M., Lamas B., Pham H.P., Michel M.-L., Rainteau D., Bridonneau C., da Costa G., van Hylckama Vlieg J., Sovran B., Chamignon C. (2018). *Bilophila wadsworthia* Aggravates High Fat Diet Induced Metabolic Dysfunctions in Mice. Nat. Commun..

[134] Peck S.C., Denger K., Burrichter A., Irwin S.M., Balskus E.P., Schleheck D. (2019). A Glycyl Radical Enzyme Enables Hydrogen Sulfide Production by the Human Intestinal Bacterium *Bilophila wadsworthia*. Proc. Natl. Acad. Sci. USA.

[135] Feng Z., Long W., Hao B., Ding D., Ma X., Zhao L., Pang X. (2017). A Human Stool-Derived *Bilophila wadsworthia* Strain Caused Systemic Inflammation in Specific-Pathogen-Free Mice. Gut Pathog..

[136] Méndez-Salazar E.O., Ortiz-López M.G., Granados-Silvestre M.d.L.Á., Palacios-González B., Menjivar M. (2018). Altered Gut Microbiota and Compositional Changes in *Firmicutes* and *Proteobacteria* in Mexican Undernourished and Obese Children. Front. Microbiol..

[137] Tang X.J., Fu R., Li Q., Liu X.Q., Hu S.F. (2021). Differential Analysis of Intestinal Flora in Children with Different Clinical Phenotypes of Henoch-Schonlein Purpura of Different Clinical Phenotypes. Chin. J. Immunol..

[138] Palmieri O., Bossa F., Castellana S., Latiano T., Carparelli S., Martino G., Mangoni M., Corritore G., Nardella M., Guerra M. (2024). Deciphering Microbial Composition in Patients with Inflammatory Bowel Disease: Implications for Therapeutic Response to Biologic Agents. Microorganisms.

[139] Obregon-Tito A.J., Tito R.Y., Metcalf J., Sankaranarayanan K., Clemente J.C., Ursell L.K., Zech Xu Z., Van Treuren W., Knight R., Gaffney P.M. (2015). Subsistence Strategies in Traditional Societies Distinguish Gut Microbiomes. Nat. Commun..

[140] Xiao Y., Cai Y., Bommineni Y.R., Fernando S.C., Prakash O., Gilliland S.E., Zhang G. (2006). Identification and Functional Characterization of Three Chicken Cathelicidins with Potent Antimicrobial Activity. J. Biol. Chem..

[141] Laviad-Shitrit S., Izhaki I., Lalzar M., Halpern M. (2019). Comparative Analysis of Intestine Microbiota of Four Wild Waterbird Species. Front. Microbiol..

[142] Ali I., Liu K., Long D., Faisal S., Hilal M.G., Ali I., Huang X., Long R. (2021). Ramadan Fasting Leads to Shifts in Human Gut Microbiota Structured by Dietary Composition. Front. Microbiol..

[143] Dam B., Misra A., Banerjee S. (2019). Role of Gut Microbiota in Combating Oxidative Stress. Oxidative Stress in Microbial Diseases.

[144] Heeney D.D., Gareau M.G., Marco M.L. (2018). Intestinal *Lactobacillus* in Health and Disease, a Driver or Just along for the Ride?. Curr. Opin. Biotechnol..

[145] Annuk H., Shchepetova J., Kullisaar T., Songisepp E., Zilmer M., Mikelsaar M. (2003). Characterization of Intestinal *Lactobacilli* as Putative Probiotic Candidates. J. Appl. Microbiol..

[146] Long Y., Peng J. (2023). Interaction between Boron and Other Elements in Plants. Genes.

[147] Arciniega-Martínez I.M., Romero-Aguilar K.S., Farfán-García E.D., García-Machorro J., Reséndiz-Albor A.A., Soriano-Ursúa M.A. (2022). Diversity of Effects Induced by Boron-Containing Compounds on Immune Response Cells and on Antibodies in Basal State. J. Trace Elem. Med. Biol..

[148] Mei S., Yang X., Guo H., Gu H., Zha L., Cai J., Li X., Liu Z., Bennett B.J., He L. (2014). A Small Amount of Dietary Carbohydrate Can Promote the HFD-Induced Insulin Resistance to a Maximal Level. PLoS ONE.

[149] Siyah P., Akgol S., Durdagi S., Kocabas F. (2021). Identification of First-in-Class Plasmodium OTU Inhibitors with Potent Anti-Malarial Activity. Biochem. J..

[150] Jacobson M.P., Pincus D.L., Rapp C.S., Day T.J.F., Honig B., Shaw D.E., Friesner R.A. (2004). A Hierarchical Approach to All-atom Protein Loop Prediction. Proteins Struct. Funct. Bioinform..

[151] Bushnell B. (2018). BBTools: A Suite of Fast, Multithreaded Bioinformatics Tools Designed for Analysis of DNA and RNA Sequence Data. Jt. Genome Inst..

[152] Boratyn G.M., Thierry-Mieg J., Thierry-Mieg D., Busby B., Madden T.L. (2019). Magic-BLAST, an Accurate RNA-Seq Aligner for Long and Short Reads. BMC Bioinform..

[153] Li H., Handsaker B., Wysoker A., Fennell T., Ruan J., Homer N., Marth G., Abecasis G., Durbin R. (2009). The Sequence Alignment/Map Format and SAMtools. Bioinformatics.

[154] Oksanen A.J., Blanchet F.G., Friendly M., Kindt R., Legendre P., Mcglinn D., Minchin P.R., Hara R.B.O., Simpson G.L., Solymos P. (2020). Vegan: Community Ecology Package, Version 2.6-8.

