# Supplementary material for: The Potential Role of Boron in the Modulation of Gut Microbiota Composition: An In Vivo Pilot Study
Source: Pharmaceuticals (Basel). 2024 Oct 6;17(10):1334. doi: 10.3390/ph17101334 (PMC11510266; doi:10.3390/ph17101334)
Supplement: Supplementary file 1 [file pharmaceuticals-17-01334-s001.zip › Supplementary_Figures.pdf]

The Potential Role of Boron in the Modulation of Gut Microbiota Composition: An In Vivo Pilot Study

Nermin Basak Sentürk [1], Burcu Kasapoglu [1,2], Eray Sahin [3], Orhan Ozcan [4], Mehmet Ozansoy [5], Muzaffer Beyza Ozansoy [6], Pinar Siyah [7], Ugur Sezerman [3,8,*] and Fikrettin Sahin [1,*]

1 Department of Genetics and Bioengineering, Faculty of Engineering, Yeditepe University, 34755 Istanbul, Turkey; basak.senturk@yeditepe.edu.tr (N.B.S.); burcu.kasapoglu@abdiibrahim.com.tr (B.K.)
2 Abdi Ibrahim Pharmaceuticals, Biotechnological Products Production Facility (AbdiBio), 34538 Istanbul, Turkey
3 Biostatistics and Bioinformatics PhD Program, Institute of Health Sciences, Acibadem Mehmet Ali Aydinlar University, 34752 Istanbul, Turkey; sahin.eray89@gmail.com
4 Cryptomicrobiology, 41400 Kocaeli, Turkey; orhn.ozcn@hotmail.com
5 Department of Physiology, International School of Medicine, Istanbul Medipol University, 34810 Istanbul, Turkey; mozansoy@medipol.edu.tr
6 Department of Physical Therapy and Rehabilitation, Faculty of Health Sciences, Fenerbahçe University, 34758 Istanbul, Turkey; beyza.ozansoy@fbu.edu.tr
7 Department of Biochemistry, School of Pharmacy, Bahçeşehir University, 34353 Istanbul, Turkey; pinar.siyah@med.bau.edu.tr
8 Department of Biostatistics and Medical Informatics, Faculty of Medicine, Acibadem Mehmet Ali Aydinlar University, 34752 Istanbul, Turkey
* Correspondence: ugur.sezerman@acibadem.edu.tr (U.S.); fsahin@yeditepe.edu.tr (F.S.)

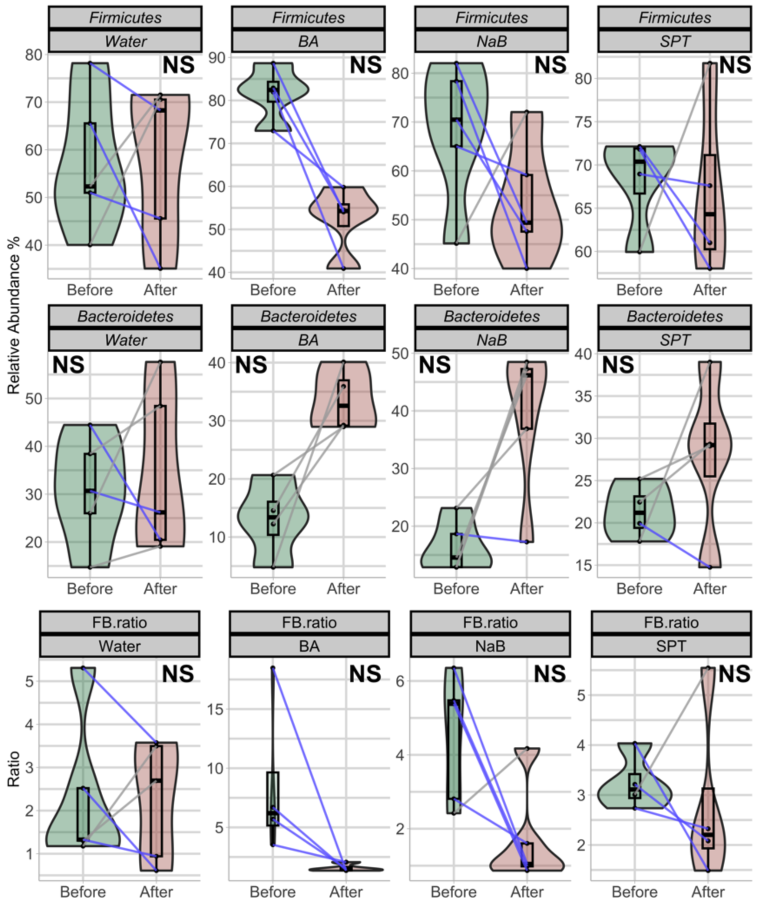

Figure S1. Time-wise changes in relative abundance of *Firmicutes*, *Bacteroidetes*, and in *Firmicutes*/*Bacteroidetes* (FB) ratio for each treatment group.

Data were represented by mixed violin and box plots for each time points. Subject-specific changes before and after treatment are shown by red, blue or gray lines to represent increased, decreased or even values, respectively. Results of significant tests were stated on the figures based on p.adj values. NS: non-significant. Water (n = 5), BA: boric acid (n = 4), NaB: sodium pentaborate pentahydrate (n = 5), SPT: sodium perborate tetrahydrate (n = 4).

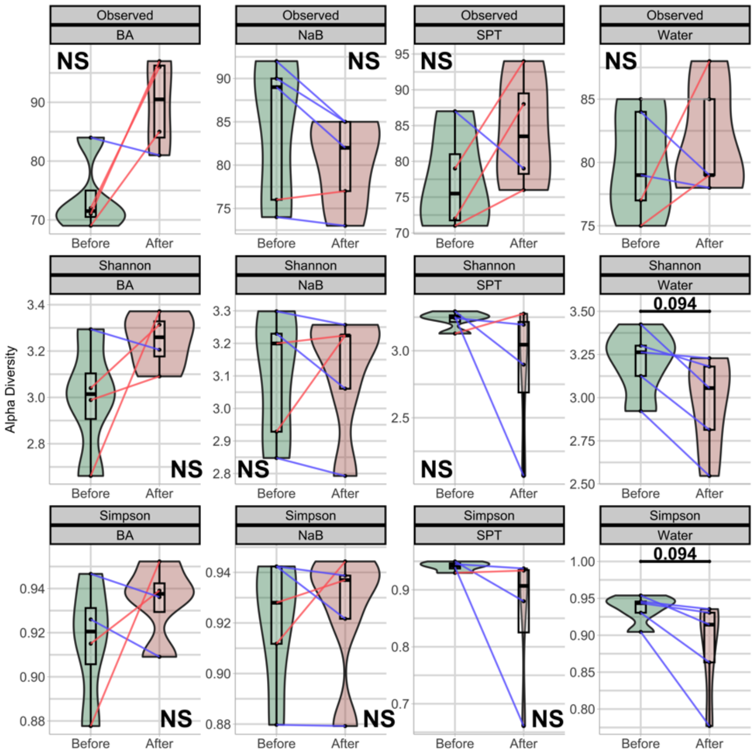

Figure S2. Time-wise changes in observed features, Shannon and Simpson indices in each treatment group for genus level.

Data were represented by mixed violin and box plots for each time points. Subject-specific changes before and after treatment are shown by red, blue or gray lines to represent increased, decreased or even values, respectively. Results of significant tests were stated on the figures based on p.adj values. NS: non-significant. Water (n = 5), BA: boric acid (n = 4), NaB: sodium pentaborate pentahydrate (n = 5), SPT: sodium perborate tetrahydrate (n = 4).

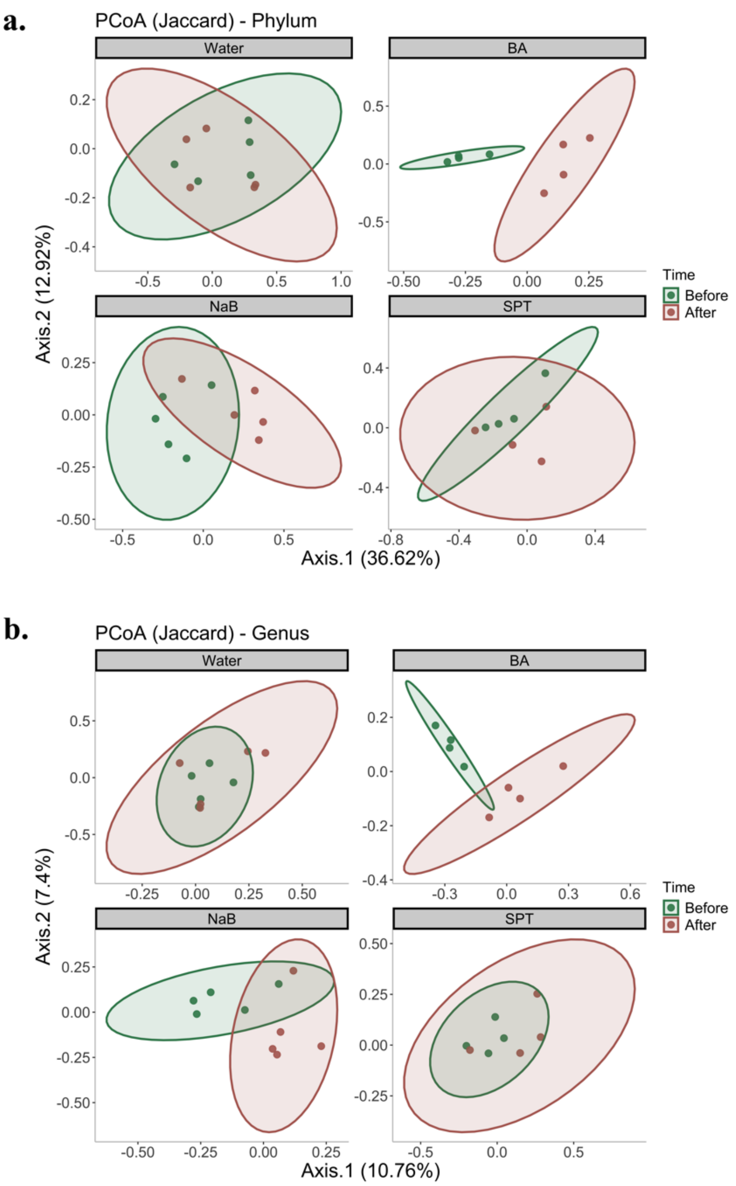

Figure S3. PCoA plots of beta diversity in (a) phylum and (b) genus level compositions based on Jaccard dissimilarity before and after treatments.

BA: boric acid, NaB: sodium pentaborate pentahydrate, SPT: sodium perborate tetrahydrate, and control group supplied with water. For beta diversity, the time points were repsented by different colours for before and after treatments. Points represent each mouse sample. Ellipses correspond to 95% confidence intervals for each of the treatment groups. Water (n = 5), BA: boric acid (n = 4), NaB: sodium pentaborate pentahydrate (n = 5), SPT: sodium perborate tetrahydrate (n = 4).

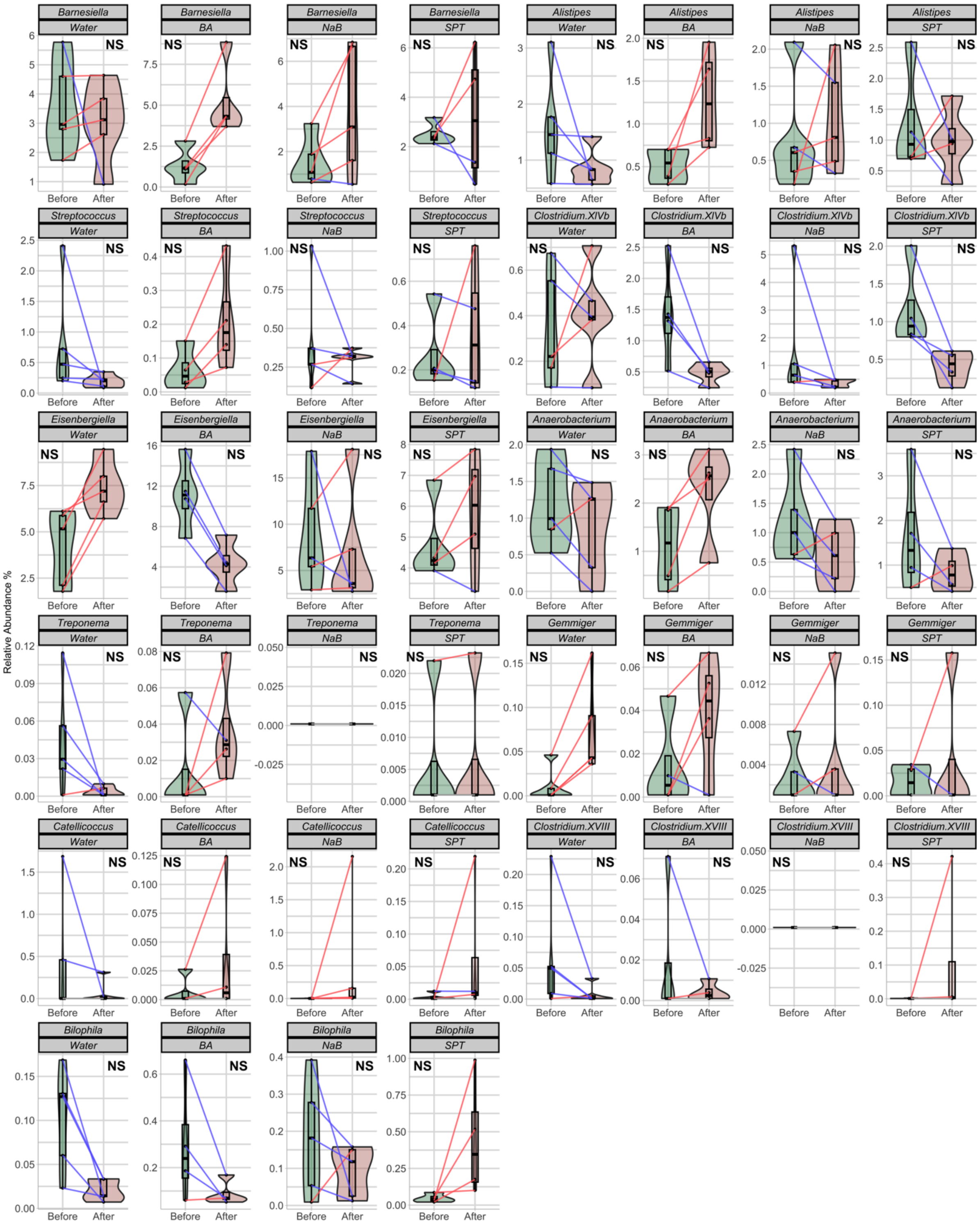

Figure S4. Time-wise changes in relative abundance of the eleven genera in each treatment group.

Data were represented by mixed violin and box plots for each time points. Subject-specific changes before and after treatment are shown by red, blue or gray lines to represent increased, decreased or even values, respectively. Results of significant tests were stated on the figures based on p.adj values. NS: non-significant. Water (n = 5), BA: boric acid (n = 4), NaB: sodium pentaborate pentahydrate (n = 5), SPT: sodium perborate tetrahydrate (n = 4).
